# *TP53* mutations, tetraploidy and homologous recombination repair defects in early stage high-grade serous ovarian cancer

**DOI:** 10.1093/nar/gkv111

**Published:** 2015-04-27

**Authors:** Jeremy Chien, Hugues Sicotte, Jian-Bing Fan, Sean Humphray, Julie M. Cunningham, Kimberly R. Kalli, Ann L. Oberg, Steven N. Hart, Ying Li, Jaime I. Davila, Saurabh Baheti, Chen Wang, Sabine Dietmann, Elizabeth J. Atkinson, Yan W. Asmann, Debra A. Bell, Takayo Ota, Yaman Tarabishy, Rui Kuang, Marina Bibikova, R. Keira Cheetham, Russell J. Grocock, Elizabeth M. Swisher, John Peden, David Bentley, Jean-Pierre A. Kocher, Scott H. Kaufmann, Lynn C. Hartmann, Viji Shridhar, Ellen L. Goode

**Affiliations:** 1 Department of Cancer Biology, University of Kansas Medical Center, Kansas City, KS 66160, USA; 2 Department of Health Sciences Research, Mayo Clinic, Rochester, MN 55905, USA; 3 Illumina, Inc., San Diego, CA 92122, USA; 4 Illumina Cambridge Ltd, Little Chesterford, Essex CB10 1, UK; 5 Department of Laboratory Medicine and Pathology, Mayo Clinic, Rochester, MN 55905, USA; 6 Department of Oncology, Mayo Clinic, Rochester, MN 55905, USA; 7 Wellcome Trust, Medical Research Council Stem Cell Institute, University of Cambridge, Cambridge CB2 1TN, UK; 8 Department of Health Sciences Research, Mayo Clinic, Jacksonville, FL 32224, USA; 9 Department of Internal Medicine, Rinku General Medical Center, Izumi-sano, 598-8577, Japan; 10 Department of Pathology and Immunology, Washington University in St. Louis, St. Louis, MO 63110, USA; 11 Department of Biomedical Informatics and Computational Biology, University of Minnesota, Minneapolis, MN 55414, USA; 12 Department of Obstetrics and Gynecology, University of Washington, Seattle, WA 98109, USA

## Abstract

To determine early somatic changes in high-grade serous ovarian cancer (HGSOC), we performed whole genome sequencing on a rare collection of 16 low stage HGSOCs. The majority showed extensive structural alterations (one had an ultramutated profile), exhibited high levels of p53 immunoreactivity, and harboured a *TP53* mutation, deletion or inactivation. *BRCA1* and *BRCA2* mutations were observed in two tumors, with nine showing evidence of a homologous recombination (HR) defect. Combined Analysis with The Cancer Genome Atlas (TCGA) indicated that low and late stage HGSOCs have similar mutation and copy number profiles. We also found evidence that deleterious *TP53* mutations are the earliest events, followed by deletions or loss of heterozygosity (LOH) of chromosomes carrying *TP53, BRCA1* or *BRCA2*. Inactivation of HR appears to be an early event, as 62.5% of tumours showed a LOH pattern suggestive of HR defects. Three tumours with the highest ploidy had little genome-wide LOH, yet one of these had a homozygous somatic frame-shift *BRCA2* mutation, suggesting that some carcinomas begin as tetraploid then descend into diploidy accompanied by genome-wide LOH. Lastly, we found evidence that structural variants (SV) cluster in HGSOC, but are absent in one ultramutated tumor, providing insights into the pathogenesis of low stage HGSOC.

## INTRODUCTION

Worldwide, ovarian cancer has been estimated to affect 225 500 women and claim 140 200 lives annually (1). The majority of ovarian cancers are of epithelial origin and consist of four major morphological subtypes: serous, endometrioid, clear cell and mucinous (2). Low-grade serous and mucinous carcinomas may develop in stepwise fashion from adenomas to carcinomas, while clear cell and endometrioid carcinomas often arise from endometriosis. In contrast, high-grade serous (HGS) carcinomas develop from an undefined precursor lesion and may progress rapidly without obvious intermediate steps (3). Due to this rapid progression, as well as the lack of specific symptoms and effective early detection methods, HGS ovarian carcinomas are the most lethal subtype, being primarily diagnosed at advanced stages (4). Consequently, early stage HGS ovarian carcinoma is rare (5). In order to elucidate early events in this lethal disease, determine whether the pathogenesis of early stage disease is the same or different from more advanced disease, and accelerate the development of genome-based biomarkers for early detection, we analyzed whole cancer genomes for 16 low stage HGS ovarian carcinomas from the Mayo Clinic and cancer exomes from 28 low stage and 316 late stage HGS carcinomas from TCGA.

## MATERIALS AND METHODS

### Mayo Clinic patients and sequencing

We characterized the whole genomes and transcriptomes from 16 low stage HGS ovarian cancer cases seen at Mayo Clinic (Supplementary Tables S1 and S13). Fresh frozen carcinomas and patient-matched blood samples were used to generate DNA paired end libraries that were sequenced using 100 bp reads on an Illumina GAIIx platform and for genotyping on Illumina Human660W-Quad BeadChip arrays. RNA sequencing was performed on 14 samples using RNA from fresh frozen cancer tissue on the Illumina GAIIx using 50 bp paired end reads. Formalin-fixed paraffin-embedded carcinomas were used for immunohistochemical staining.

### TCGA data

In additional somatic mutation analyses, we utilized exome sequence data from 28 low stage HGS carcinomas and 316 late stage HGS carcinomas from the TCGA (6). For additional copy number (CN) analysis, we utilized 28 low stage HGS carcinomas and 446 late stage HGS carcinomas with access-level 3 segmented data from Affymetrix 6.0 single nucleotide polymorphism (SNP) arrays (downloaded from tcga-data.nci.nih.gov).

### Bioinformatic sequence analysis

To call DNA sequence variants using single samples, genotypes were initially called on carcinoma and germline samples separately using the Casava 1.7 pipeline. Those calls were compared to the genotype calls from Illumina Human660W-Quad BeadChip. For somatic paired variant calling, reads were aligned with the bwa (7) aligner, then realigned as paired samples using the IndelRealigner in GATK v0.6 (8). Somatic variants were called with SomaticSniper (SS) v1.0 (9) for single nucleotide variant (SNV) calls and GATK Somatic Indel detector in GATK v0.6 (8) for insertions/deletions (indels). Variants were annotated using the TREAT workflow (10). Variants were filtered, requiring a minimum somatic score of 15 for SNV, not overlapping a SNP/indel in dbSNP132 (with a minor allele frequency (MAF) of 1% or more), at least two mutant alleles in the carcinoma (with quality of at least Q20), with one forward and one reverse read, maximum fisher strand bias of 40, no more than one mutant read in the germline (or 1% of coverage, whichever is higher) and with no more than two highly homologous regions in the human genome (85% homology using blat) for a region with 50 nucleotides on either side of the variant. To identify significant somatic mutations (i.e. enriched beyond regional background mutation rates), we used the background mutation rate computed with bmr and smg tools of GenomeMuSiC package v0.4 (11). To count transcript levels in RNA sequence data, 50 bp paired end reads were aligned with against hg19 using Tophat 1.4 (12), with the option to align first to known genes with bowtie 1; counts were produced using htseq 0.5.3 (13).

### Technical validation of Mayo Clinic somatic mutations

Five carcinomas were sequenced on a standardized NextGen sequencing cancer panel (Supplementary Table S2) at key genes of interest; all carcinomas with *TP53* and *BRCA2* mutations were confirmed by this approach. Candidate somatic or deleterious germline mutations were filtered from the standardized NextGen panel results by removing common SNPs (MAF 2% of higher in dbSNP 137) as long as they did not have known deleterious alleles in dbSNP or a locus-specific database (14). Remaining variants are reported in Supplementary Table S3. All the somatic variants reported by NextGen sequencing were also included in the filtered set of somatic variants from the whole genome sequencing.

### Mutation validation with Sanger sequencing

Sanger sequencing of regions containing somatic mutations in the 20 most frequently mutated genes among 16 tumors was performed using dye termination chemistry (Big Dye Terminator with the model 3730xl sequencer; Applied Biosystems, CA). Primer sets for polymerase chain reaction (PCR) were designed using the design tool Oligo 6.0. PCR was carried out using AmpliTaq Gold DNA Polymerase (Applied Biosystems) based on the standard protocol. Amplicons from target genes in 16 tumors were amplified (Supplementary Table S4). After PCR, amplicons were treated with the ExoSAP-IT (USB Corp, OH) to degrade unincorporated PCR primers and deoxynucleotide triphosphates. The cleaned products were mixed with 5 pmol of the forward or reverse PCR primers for sequencing. DNA sequence variants were identified using Mutation Surveyor software.

### Mutation validation with RNA sequencing and qRT-PCR data

Fourteen out of the 16 Mayo Clinic cancer samples were also subjected to RNA-sequencing analysis as a secondary confirmation of somatic variant calls. TopHat 1.4 was used for alignment to human reference genome (hg19) and variant reporting was done using GATK at the locations of DNA mutations. Additionally, for *TP53*, manual review of the variants was performed using Integrative Genomics Viewer (IGV). Validation provided by RNA was used to estimate sensitivity and specificity of the DNA somatic calls. Quantitative Reverse Transcriptase PCR (qRT-PCR) was also used to validate mutations.

### Sensitivity and specificity of somatic variant calling pipeline

Because the variants that were Sanger sequenced were generated by an independent pipeline, we estimated the sensitivity/specificity of our calling strategy. Estimating the truth for variants using Sanger sequencing results will be a pessimistic evaluation because Sanger sequencing is not sensitive for heterozygous mutations in tumor with low tumor content or with low percentage subclones. A number of mutations have convincing ‘high confidence NextGen’ variants (mapping quality 30, base quality 30, no overlap with repeats, genome duplications or homologous regions) with negative results in Sanger sequencing but mean mutant allele frequency (MMAF) of only 31%, perhaps because they were in subclones. Half of those high-confidence variants had confirmatory RNASeq alleles, indicating that perhaps they are below the percentage detection threshold of Sanger sequencing. Depending on how the true positive mutations are defined, our sensitivity/specificity can be estimated as 76%/36% (strictly trusting Sanger Results), 84%/55% (high confidence next gen +RNASeq) and 94%/100% (or trusting NextGen results over negatives in Sanger Sequencing). Only three variants (6%) that ended up validating were filtered out by our filtering strategy. The number of indels that were Sanger sequenced was too small to evaluate sensitivity/specificity, but the sensitivity should be poor with standard GATK parameters (requires the indel be present in at least 30% of reads).

### p53 immunohistochemistry

Sections of Mayo Clinic tissues were deparaffinized, rehydrated and submitted to antigen retrieval by a steamer for 25 min in target retrieval solution (Dako, Carpinteria, CA, USA). Endogenous peroxide was diminished with 3% H_2_O_2_ for 30 min. Slides were blocked in protein block solution for 30 min and then blocked with avidin and biotin for 10 min each, followed by overnight incubation with 1:50 diluted anti-p53 antibody (M7001, Clone DO-7, DAKO) at 4°C. Sections were then incubated with biotinylated universal link for 15 min and streptavidin-horseradish peroxidase for 25 min at 25°C. Slides were developed in diaminobenzidine and counterstained with hematoxylin as previously described (15).

### Copy number estimation

The CN in cancer genomes from the Mayo Clinic patients was estimated using two approaches. First, whole genome CN was computed using a novel algorithm (patternCNV) which is able to use depth of coverage to compute CN variation despite local coverage variation by learning the pattern of this coverage bias (16). PatternCNV firstly summarizes coverage variation across multiple germline samples to evaluate the local coverage and the variation. Somatic CN were computed for each sample as the difference from the reference pattern in windows of 5000 nucleotides. Regions of CN were computed using the DNAcopy package by weighting each bin inversely to the variance of the pattern. Second, Illumina 650 genotyping array data were used. We used the paired option in Illumina GenomeStudio and extracted the log relative ratio (LRR) and the B-allele frequency of the germline and tumor DNA. The genoCN (17) R package was used to detect somatic CN alterations (CNAs) requiring a minimum of 20 probes per CNA interval. The genoCNA function also estimates computationally the tumor purity, defined as the percentage of cancer cells in the tissue specimen (Supplementary Table S5), based on the levels of the CN segments.

### Ploidy and LOH estimation

The WaveCNV (18) segmenter was used to compute loss of heterozygosity (LOH) and ploidy genome wide using the pattern of alleles and CN. As input to the tool, we provided (i) A Variant Calling Format (VCF) file containing the number of reads carrying alleles for germline variants that were called using GATK 2.6 in discovery mode for paired samples, (ii) CN segments obtained from PatternCNV and (iii) known tumor purity from cellularity measurements. The program outputs ploidy, CN per segment and LOH_region calls.

### CN comparisons

To minimize false detection of CNAs, we limited analysis to recurrent regions identified in TCGA carcinomas which were previously reported to be recurrent (19,20). The aim was to find CN regions differing in prevalence between low stage and high stage cases. In the first step, we used data sets consisting of SNP-based CN estimation (15 Mayo Clinic low stage cases excluding a detected ultramutated carcinoma; 28 TCGA low stage cases; 446 TCGA late stage cases) and WGS-based CN estimation (15 Mayo Clinic low stage cases excluding a detected ultramutated carcinoma). Given the *k*th region and a CN cutoff, we compute CN occurrence frequency *f_k_*, _late_ in late stage, and check if *f_k_*, _TCGA-early_, *f_k_*, _Mayo-early-SNP_ and *f_k_*_, Mayo-early-SNP_ have consistent and significant difference with *f_k_*_, late_, based on binomial test. Array comparative genomic hybridization (aCGH) data from TCGA serous was downloaded as level 3 data. Segmented data log2 ratios were then averaged over CN region of interest. Average log2 ratios were compared between the low versus late stage carcinomas using a *t*-test.

### Temporal relationship between somatic mutation and CN in Mayo Clinic patients

For every gene with recurrent somatic mutations (more than one mutation observed in Mayo Clinic low stage HGS cases), we identified somatic SNVs (deleterious or benign) within 1 Mb of each gene. These somatic SNVs were filtered to have at least 15 reads in the tumor (so we can accurately estimate the frequency of mutations), no mutant allele in the germline, not overlapping a dbSNP SNP and with germline coverage of at least 10. The somatic frequency for that range was defined as the weighted mean of individual SNV frequencies, weighted by the one minus the probability that this could be a SNP given the germline coverage. The CN was a segment of constant value for the selected region, but was computed as the mean across all somatic SNVs. To correct for low purity (percentage of tumor in specimen), we divided the log2 ratio by the global tumor purity estimated from all variants. We expected the MMAF *m* for a deletion in clone present in a fraction *c_i_* of the tumor in a sample with purity *x* to be m = x*c_i_/((2*(1−c_i_*x)) + 1*x*c_i_). If all mutations in that region occurred before deletion events, *m* should be given by this formula. If some mutation occurred after deletion events, *m* will be reduced by the fraction *f_i_* of the clone for sample *i* carrying the mutation (Supplementary Figure S5)

(1) \begin{equation*} m = x*c_i *f_i /((2*(1 - c_i *x)) + 1*x*c_i. \end{equation*}

With each purity-corrected CNA *= c_i_x*, we computed a mean estimator *F* for the low stage/late stage factor as

(2) \begin{equation*} F = < f_i >= \frac{1}{n}\sum\nolimits_{i = 1}^n {(m - cna)\left( {\frac{{2*(1 - cna)}}{{cna}}} \right).} \end{equation*}

We estimated the minimum and maximum confidence range using leave-one-out cross validation. In Supplementary Figure S1, the red line is the data fit, while the blue lines are the fit from the leave-one-out cross validation data sets. A one-sided Wilcoxon test of *f_i_*-1 is used to estimate the significance of the results when *F*<1.

### Kataegis analysis

Mutations were additionally filtered to remove mutations that are potentially from mapping artifact by removing any mutations where a 100 bp region surrounding the mutation maps at more than three locations on the genome at 90% identity using Basic Local Alignment Search Tool. The graph was generated to plots the distance between consecutive mutations on a sample. Pink and black lines at the bottom alternate color between different chromosomes (even and odd chromosomes), and vertical dotted green lines highlight chromosome boundaries.

### HR-deficient signatures

The signature from (21) was used to create a heatmap. Because the signature was created on a different platform, we only selected genes with the top 50% of expression in our data set and a ratio of standard deviation to median expression in the top 50%. Gene counts were divided by the total gene counts of each sample, and the median was subtracted prior to dividing by the standard deviation. Hierarchical clustering of the samples was performed in R using the heatmap function with the Euclidean distance metric. Results are shown in Supplementary Figure S2.

### Structural variant analyses

Structural variants (SVs) were called using CREST 1.0 (22), SVs where the ends were within 10 bp of each other were marked as duplicate and only counted once. SVs within 1 Mb of each other were considered clustered.

## RESULTS

From 16 low stage HGS ovarian cancer cases treated at the Mayo Clinic, we generated an average of 46-fold coverage of the haploid genomes (tumor and matched germline DNA) by whole genome sequencing. Approximately 93% of the exomes and 86% of the genomes were covered by at least 10 reads in both tumor and germline DNA (Supplementary Table S1). Concordance between sequencing SNVs and SNP array genotypes was greater than 97% (Supplementary Table S6). In total, we identified 138 767 somatic single nucleotide mutations among 15 low stage HGS carcinomas (Table 1) and 1 087 366 somatic single nucleotide mutations in an ultramutated low stage cancer. The ultramutated stage IC HGS carcinoma was obtained from a patient not exposed to prior chemotherapy or radiation therapy and without any previous cancer diagnosis. This ultramutated carcinoma had a *POLE* V411L mutation previously implicated in ultramutated endometrial (23,24) and colorectal carcinomas (25). Excluding this carcinoma, there were a total of 949 coding or splice-site somatic mutations among the remaining 15 low stage HGS ovarian carcinomas. From this point forward, analyses excluded the ultramutated carcinoma unless otherwise stated.

**Table 1:** Count of somatic mutations per patient by mutation type

	SNV annotation									INDEL annotation									
Patient	3′Flank	Missense	5′UTR	Nonsense	Intergenic	Splice site	Silent	5′Flank	*De novo* start InFrame	3′UTR	Intron	3′Flank	3′UTR	5′Flank	Frame shift del	Frame shift ins	Intergenic	Intron	Total per patient
A	199	25	3	2	4309	1	13	138	1	25	2171	16	2	10	2	0	265	161	7343
B	88	16	1	2	2965	0	2	75	0	17	1392	5	0	8	0	0	114	48	4733
C	178	26	3	1	4104	0	9	150	0	35	1892	14	1	14	1	1	263	146	6838
D	23474	5092	217	453	638744	103	1963	17989	73	5316	338317	1385	311	1101	66	37	33156	19569	1087366
E	303	59	8	1	6855	1	17	212	1	45	3541	13	2	16	0	0	263	123	11460
F	196	25	4	2	4252	2	14	135	2	24	2104	32	3	12	4	2	371	196	7380
G	304	61	3	4	6625	3	23	229	1	51	3447	29	6	20	3	1	327	235	11372
H	47	4	0	1	1239	1	5	40	0	8	611	4	2	3	1	2	80	62	2110
I	141	18	4	2	3560	1	12	128	0	19	1778	24	1	17	3	2	294	190	6194
J	173	29	3	2	4253	1	13	152	2	46	2270	24	1	11	6	1	340	179	7506
K	259	24	4	1	4448	1	15	175	0	20	2338	26	1	21	0	4	282	198	7817
L	141	23	2	1	3587	1	9	120	0	15	1768	10	2	7	3	0	173	109	5971
M	477	86	2	6	9883	7	39	382	1	68	5030	32	1	26	5	1	615	350	17011
N	371	75	5	6	10227	1	22	329	3	62	4621	52	5	29	3	0	886	443	17140
O	133	21	0	1	3893	1	11	131	0	14	1803	16	2	10	0	1	188	105	6330
P	478	97	10	6	11968	2	35	366	1	74	5944	25	2	11	1	1	345	196	19562
Total without Patient D	3488	589	52	38	82168	23	239	2762	12	523	40710	322	31	215	32	16	4806	2741	138767
Total unique coding without Patient D: 949																			
Total unique non-coding without Patient D: 129128																			

Summary of mutations by region and functional classification according to SNPEFF. Note that the cancer from Patient D had an ultramutated phenotype.

**Figure 1.**
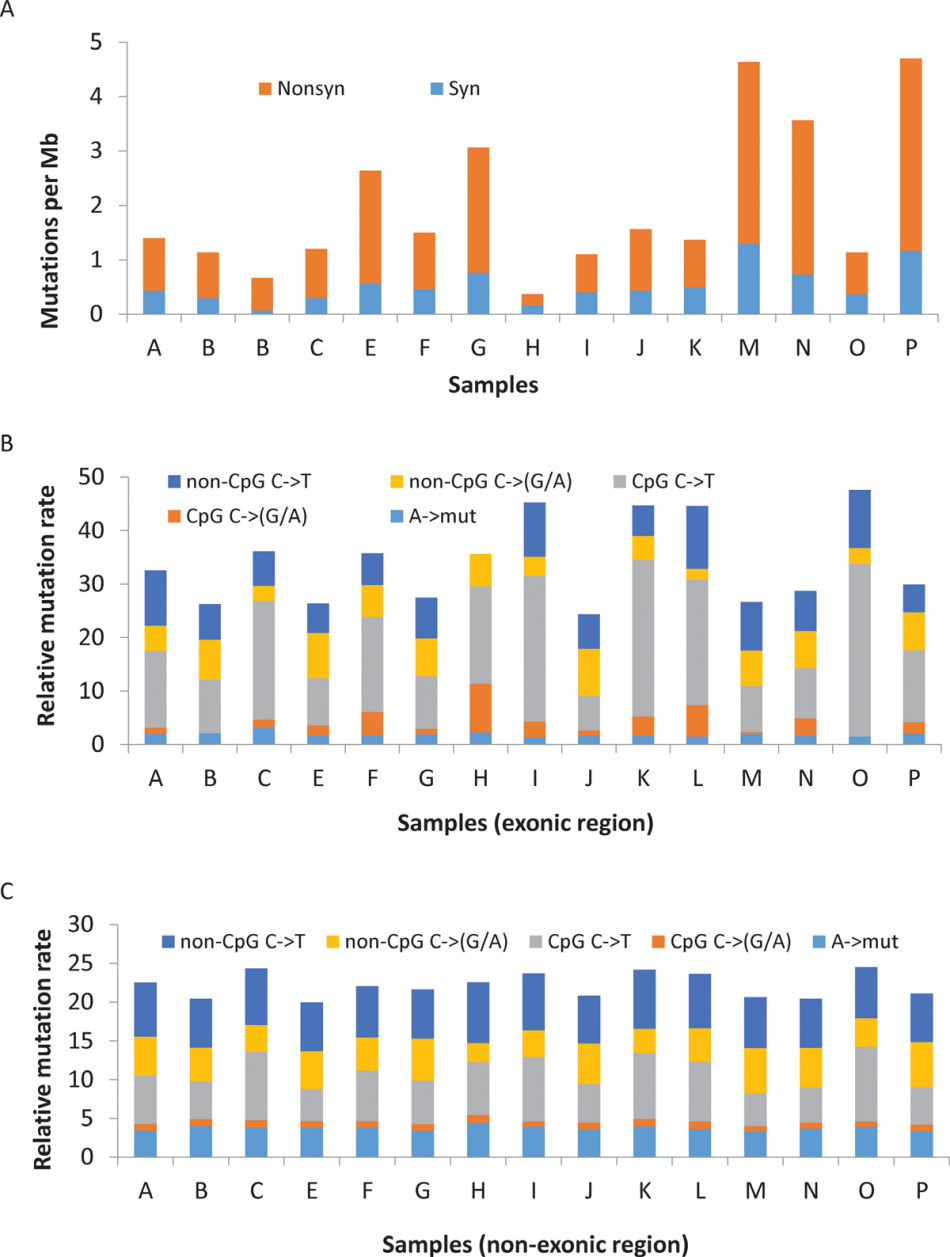
Somatic mutation rates in early stage ovarian cancer genomes. (**A**) Rate of synonymous and non-synonymous mutations in 15 early stage ovarian cancer genomes. (**B** and **C**) Mutation rate for selected types of mutation (indicating nucleotide before mutated nucleotide, nucleotide to mutate and possible product of mutation) for each cancer, divided by the number of mutational contexts for each mutation type, in order to detect possible biases for mutational processes (e.g. CpG->(G/A) has two contexts) in exonic regions (B) and non-exonic regions (C). Note that C>T somatic substitutions are significantly higher at CpG sites than at non-CpG sites in coding regions (B) but not in non-coding regions (C). A>mut is the mutation rate of A to any other substitutions.

Among the 15 low stage HGS cases, there were a greater number of non-synonymous than synonymous somatic mutations (paired *t*-test, *P*<1.1×10^−4^) (Figure 1A), with a mean ratio of 2.89; this was also true for the ultramutated cancer. C to T (C>T) transitions were the most common mutations (27%) and were enriched relative to the mean number of mutations per type (*P* = 7.0×10^−8^). The least common mutations were T>G transversions (8%) (Supplementary Table S7), which were rarer than expected by chance (*P* = 8.0×10^−6^). Enrichment of C>T at CpG sites was different in exonic versus non-exonic regions. Within the exonic regions, C>T substitutions were significantly higher at CpG sites than at non-CpG sites (paired *t*-test, *P* = 2.5×10^−4^) (Figure 1B). High rates of C>T substitutions at CpG sites were also observed in previous tumor exome studies (26,27); the deamination of methylated Cs has been suggested as a possible mechanism of C>T substitutions. In contrast, in non-exonic regions, C>T substitution rates at CpG and non-CpG sites were similar (*P* = 0.19) (Figure 1C), consistent with global hypomethylation in cancers (28).

**Figure 2.**
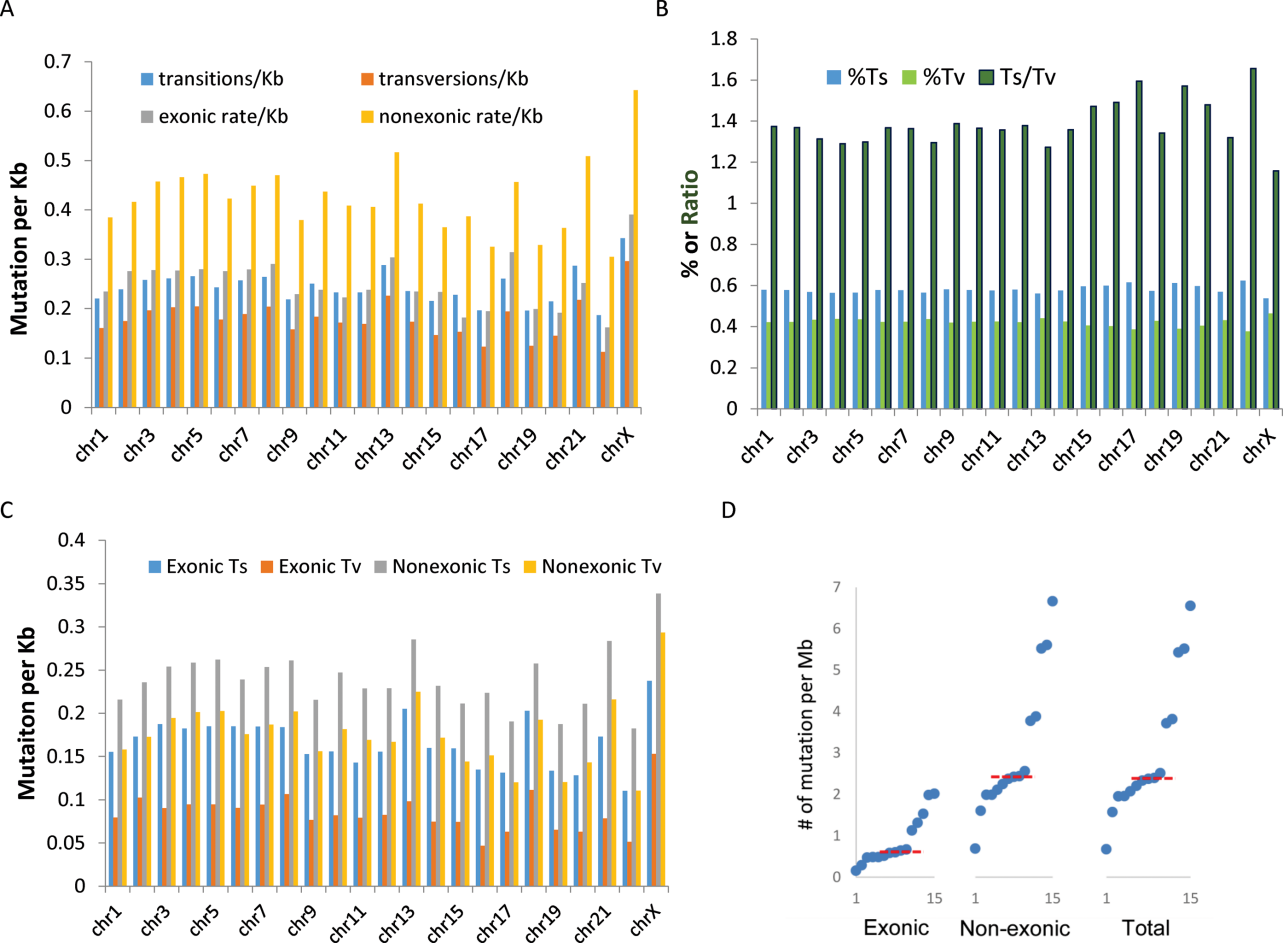
Somatic mutation patterns in early stage ovarian cancer genomes. (**A**) Total transition and transversion rates (across 15 carcinomas, divided by 15 to get per patient rates) for all regions within each chromosome and mean mutation rates in coding and non-coding regions within each chromosome. (**B**) Fraction of transition (%Ts), transversion (%Tv) and the ratio of Ts/Tv for each chromosome. (**C**) Total transition and transversion rates within coding and non-coding regions of each chromosome. (**D**) Mutation rates within coding (exonic), non-coding (non-exonic) regions for each cancer on the *x*-axis. Median is indicated by red dotted line. Note that mutation rates in non-coding regions are consistently higher than mutation rates within coding regions.

Most chromosomes showed similar mutation rates, and all showed greater transition than transversion rates (Figure 2A). Chromosomes 17, 19 and 22 showed significantly higher genomic (exonic + non-exonic) transition/transversion ratios compared to the average (paired *t*-test, *P*-values 0.03, 0.048 and 0.007, respectively) (Figure 2B), which may reflect the fact that these chromosomes have the highest GC content. These chromosomes also had the lowest non-exonic mutation rates (Figure 2C). Chromosome X had significantly lower transition/transversion ratios than the genome-wide average (*P* = 0.0198) (Figure 2B). The ultramutated cancer (Patient D) also had more transitions than transversions (Supplementary Table S7). Genome wide, the mutation rate was significantly higher in the non-exonic regions compared to the exome (paired *t*-test, *P* = 2.0×10^−17^) (Figure 2D), suggesting some selection against mutations in exonic regions.

**Figure 3.**
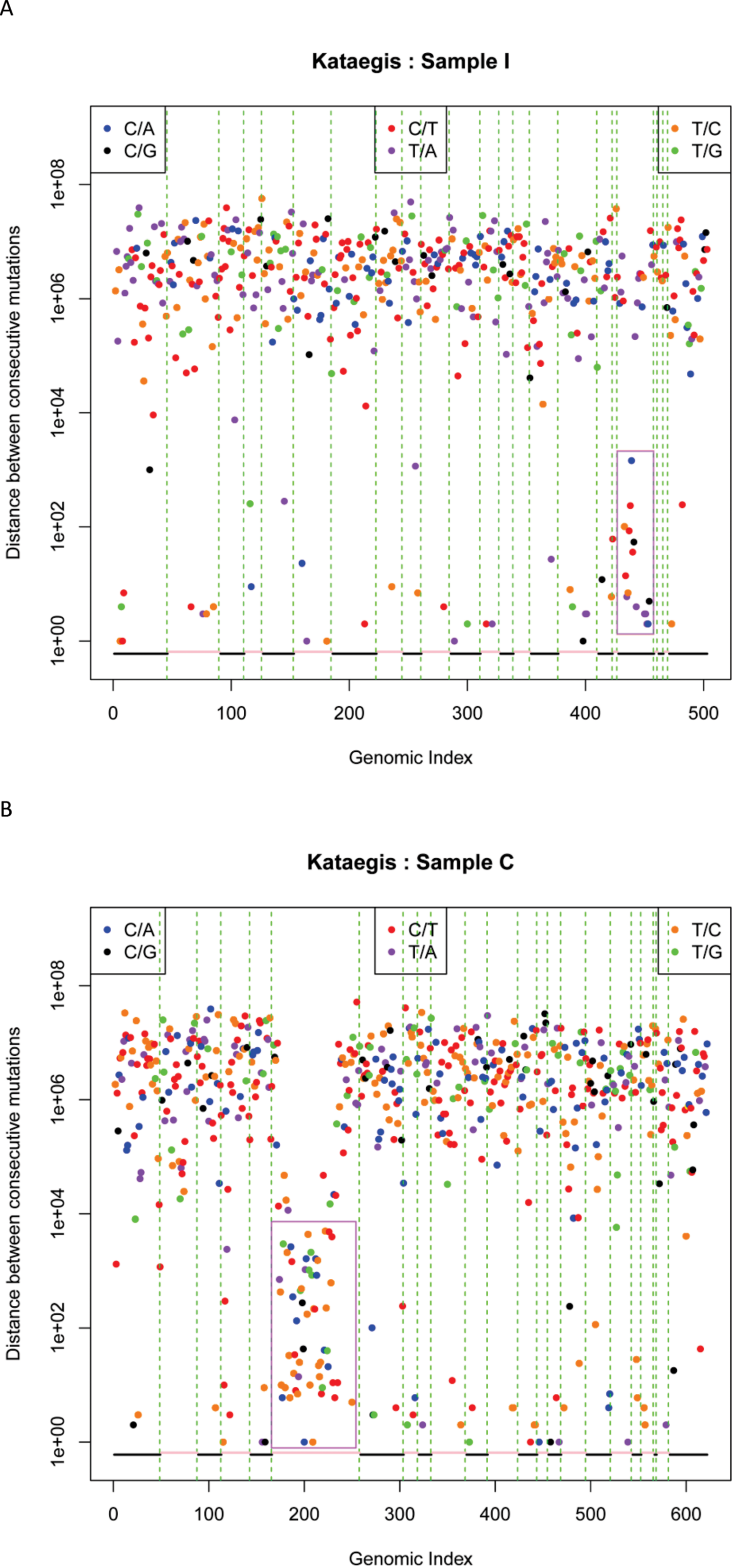
Kataegis events in early stage ovarian cancer genomes. (**A**) Patient I. (**B**) Patient C. The ‘rainfall’ plots represent total mutational events from individual carcinomas, and each dot represents a single somatic mutation plotted on the horizontal axis corresponding to its order in the human genome. Regions of interest with kataegis events are shown in purple boxes.

Recent studies have uncovered the presence of hypermutated regions, termed kataegis, in some cancer genomes (29). Therefore, we investigated evidence for kataegis among the 16 low stage HGS ovarian carcinomas. We found evidence of a potential kataegis event in chromosome 19 in Patient I, a stage I ovarian cancer (Figure 3A). In addition, we found a novel chromosome-wide hypermutational event with enriched T>C substitutions in chromosome 6 in the cancer from Patient C with stage IB disease (Figure 3B). This observation is novel because previous kataegis events were associated with either C>T or C>G substitutions (29).

Excluding the ultramutated cancer, the coding sequences of 38 genes were mutated in more than one of the 15 low stage HGS ovarian carcinomas and were above the expected background mutation rates corrected for gene size and specific mutation rate (Table 2). Manual curation of mapped sequences using IGV (30,31) revealed that the *TP53* gene was mutated in all but one of the Mayo Clinic low stage HGS ovarian carcinomas (Table 3) and at high mutant frequency when results in these carcinomas were combined with the 28 low stage HGS carcinomas of TCGA (Supplementary Table S8). A high frequency of mutations was found throughout the *TP53* coding regions as well as at known hotspots, consistent with the mutation profiles of tumor suppressor genes (26,32). Consistent with the presence of *TP53* mutations, high levels of p53 staining were observed in all but two cases analyzed (Table 3 and Supplementary Figure S3). These data are consistent with results of *TP53* studies in late stage (6,33) and low stage HGS ovarian carcinomas (34). Combined with the Mayo Clinic data, results collectively support the hypothesis that mutations in *TP53* represent the earliest documented genetic lesions in HGS ovarian cancer (34). For a subset of 14 patients with RNA sequence data (Table 3), the mutant allele was observed at a higher frequency than the wild type allele, either through deletion of the wild type allele or through epigenetic silencing of the other allele by mechanisms that were not further characterized here. The cancer from Patient H is the only with no mutation in *TP53*; this cancer, however, had a mutation in the *CHEK2* (p.V246L) gene, although the functional significance of this somatic mutation is not known .

**Table 2.** Significantly mutated genes in early stage HGS ovarian cancer from 15 Mayo Clinic patients

Gene	Indels	SNVs	Covered bps	Mutations per Mb	*P*-value
*TP53*	4	6	61 321	163.08	<10E−10
*TPSAB1*	0	3	7 483	400.91	0.0000381
*FOXD4L1*	0	2	16 900	118.34	0.017
*XIRP2*	1	2	196 902	15.24	0.017
*VEGFC*	0	2	29 078	68.78	0.047
*FAM55D*	0	2	37 974	52.67	0.054
*ASCC3*	0	3	194 324	15.44	0.035
*GPR179*	0	3	130 368	23.01	0.067
*AMIGO2*	0	2	55 035	36.34	0.073
*NUP210L*	0	2	130 499	15.33	0.084
*CDSN*	0	2	39 312	50.88	0.087
*P2RY13*	0	2	44 273	45.17	0.115
*SLC5A7*	0	2	82 795	24.16	0.127
*CHRM3*	0	2	39 835	50.21	0.136
*DYNC1I1*	0	2	70 529	28.36	0.163
*ANUBL1*	0	2	69 303	28.86	0.173
*LRRC2*	0	2	84 448	23.68	0.210
*CACNA2D1*	0	2	119 338	16.76	0.192
*BRCA2*	0	2	198 810	10.06	0.296
*UNC5D*	0	2	133 980	14.93	0.330
*MUC16*	0	3	796 308	3.77	0.210
*CC2D2A*	0	2	145 597	13.74	0.350
*SHROOM2*	0	2	114 872	17.41	0.355
*MUC2*	0	2	155 671	12.85	0.331
*MYO16*	0	2	165 213	12.11	0.357
*MUC5B*	0	2	208 010	9.61	0.353
*PKHD1L1*	0	2	288 344	6.94	0.360
*PIK3C2B*	0	2	164 801	12.14	0.440
*MLL3*	0	2	313 318	6.38	0.352
*IGFN1*	0	2	202 275	9.89	0.554
*DNAH7*	0	2	288 837	6.92	0.520

Significantly mutated genes using genome music in Mayo Clinic samples. Only genes with at least two mutations are shown; Indels, number of insertion/deletion variants in the region/gene; SNVs, number of single nucleotide variants (SNV) in the region/gene; Total mutations, total number of SNVs and Indels; Covered bps, gene size in base pairs; Mutations per Mb, mutations per Megabase; *P*-value is from Fisher's combined *P*-value test; additional mutations were found in the following genes which are members of gene families or highly polymorphic *HLA-DRB1, HLA-DQA1, OR2A14, MUC4, KRTAP4–7, OR4A47* and *TTN*.

**Table 3.** *TP53* mutations in early stage HGS ovarian carcinomas

Patient	Mutation	Mutant/total allele count	Sanger validation result	IHC staining	% Mutant allele in RNASeq	RNASeq gene count	CNV log2 ratio	Inferred non-mutated allele status
A	chr17:7578208:T->C(H214R)	12/19	Validated	Intense	85%	571	−0.39	Non-mutant allele deleted/LOH
B	chr17:7574030–7574030:delC(*fs)**	6/37	Validated	Intense	6% (52%***)	395	0.02	Non-mutant allele transcriptionally inactivated
C	chr17:7578406:C->T(R175H)	26/32	Validated****	Intense	74	908	−0.6	Non-mutant allele deleted/LOH
D	chr17:7578226:T>C(P85R) **	5/16	Validated	ND	ND	ND	−0.20	ND
E	chr17:7577568:C->A(C238F)	11/28	Validated	Intense	93%	1144	−0.34	Non-mutant allele transcriptionally inactivated
F	chr17:7578263:G->A(Stop)	20/32	Not validated****	NA	30%	174	−0.49	Non-mutant allele deleted and transcript carrying mutation degraded
G	chr17:7577556:C->A(C242F) *	22/38	Validated	Intense	94%	2459	0.06	Non-mutant allele deleted/ copy neutral LOH
H	No mutation after manual review	NA	NA	Focal	ND	ND	0.1	No Mutation
I	chr17:7577500–7577500:TG->T(*fs)	6/8	Validated	None	5% (77%***)	385	−0.70	Non-mutant allele deleted/LOH
J	chr17:7573994–7573994:TC->T(*fs)	28/30	Validated	Intense	100%	3391	0	Non-mutant allele deleted/ copy neutral LOH
K	chr17:7577538:C->T(R248Q) *	6/20	Validated	Intense	80%	990	−0.34	Non-mutant allele transcriptionally inactivated
L	chr17:7574003–7574003:delG(*fs)	15/21	Validated****	Intense	41% (74%***)	566	−0.50	Non-mutant allele deleted/LOH
M	chr17:7577062–7577062:delT(*fs)	40/46	Validated****	Intense	41% (75%***)	281	−0.21	Non-mutant allele deleted/ copy neutral LOH
N	chr17:7577121:G-:A(R273C)**	52/62	Validated	Intense	96%	2094	0.28	Non-mutant allele deleted/ copy neutral LOH
O	chr17:7577539:G->A(R248W)	18/19	Validated	Intense	86%	1839	−0.30	Non-mutant allele deleted and ploidy Amplification of mutated allele.
P	chr17:7577545:T->C(246M/V)	12/27	Validated****	Intense	80%	880	−0.35	Non-mutant allele transcriptionally inactivated

*Mutation filtered out for significance analysis.

**Mutation identified by manual review.

***Mutations with Indel or near splice site are hard to align in RNA; percentage in parentheses assumes missing sequence coverage, compared to average, should be attributed to mutant allele; ND: not able to determine.

****Also validated by next-generation sequencing.

ND: not determined.

NA, not applicable.

In addition to *TP53*, there were additional alterations in genes frequently mutated in late stage HGS (Table 4). In particular, our low stage cases contained potentially deleterious somatic variants of unknown significance (VUSs) in *BRCA2* (Patient F: P2612S and Patient M: K2017I) and *CSMD3* (Patient K: P1653Q). Moreover, one case (Patient P) had a germline frameshift mutation in exon 13 of *BRCA2* mutation along with somatic loss of the non-mutated allele in the cancer. Patients C, D, F and G also have germline VUSs in *BRCA2*. For Patients F and M, with somatic *BRCA2* mutations, the mutant *BRCA2* allele was homozygous (Table 4) as often occurs in functional mutation of tumor suppressor genes; however, both variants are VUSs. In addition, Patient N acquired a homozygous deleterious somatic mutation in *BRCA1* (Frameshift in exon 17, rs80357572) (36 out of 43 reads carried mutation), and the ultramutated carcinoma (Patient D) acquired a VUS in *BRCA1* (T231N). Patient B had a germline VUS in *BRCA1* (E1167K, rs80356923), but with predicted deleterious effects.

**Table 4.** Evidence suggestive of defects in the homologous recombination repair pathway

	RNASeq gene Counts				CNV (log2)										
Patient	*BRCA1*	*BRCA2*	*RAD51C*	*RAD51D*	*BRCA1*	*BRCA2*	*RAD51C*	*RAD51D*	Mutation	LOH (*BRCA1/2*)	CNLOH	Candidate HR defect	>15 MB	HRD score	LOH burden
A	16	38	103	48	−0.39	−0.44	−0.39	0	None	*BRCA1/2*	N	*BRCA1/2* (E)	31	27	0.43
B	145	103	130	109	−0.08	−0.11	−0.06	−0.06	*BRCA1* (G:E1167K Het->Het)	*BRCA1/2*	N	NA	11	36	0.68
C	387	347	207	132	−0.16	0.13	−0.2	−0.16	*BRCA2* (G:T1414M, G:D1902N)	*BRCA1*	*BRCA1*	NA	30	21	0.33
D	NA	NA	NA	NA	−0.23	0.03	−0.14	−0.11	*BRCA1* (S:T231N Het),multiple *BRCA2* VUS	N(*BRCA1*-W)	N	NA	13	4	0.39
E	151	162	76	38	−0.32	0.12	−0.38	−0.37	None	N	N	*RAD51D* (E)	17	0	0
F	496	222	230	77	−0.47	−0.49	0.28	−0.47	*BRCA2* (G:K3083N, S:P2612S Het->Hom)	*BRCA1/2*	N	*BRCA2* Somatic P2612S Hom (VUS)	25	20	0.33
G	35	278	129	109	−0.47	0.06	−0.17	−0.47	*BRCA2* (G:P451Q Het->Lowered to LOH)	*BRCA1*-germline (W-*BRCA1*) *BRCA2*	*BRCA2*	*BRCA1* (E) *BRCA2* (G:P451Q; Het->Hom)	50	22	0.31
H	NA	NA	NA	NA	0.11	−0.01	0	0.07	*CHEK2* (G:V246L Het->Het)	*BRCA1*	N	*CHEK2* (G:V246L Het->Het)	12	26	0.54
I	150	226	65	77	−0.78	0.14	−0.71	−0.73	None	*BRCA1*	N	*BRCA1* (C) *RAD51C* (E,C) *RAD51D* (C)	25	15	0.21
J	285	255	135	52	−0.78	−0.75	0.04	−0.78	None	*BRCA1/2*	N	*BRCA1/2* (C,L)	44	20	0.34
K	121	270	157	91	−0.23	0.51	−0.09	−0.16	NA	N	N	NA	25	14	0.25
L	404	239	142	74	0	−0.06	0	−0.47	*RAD51C* (G:T287A) lost by LOH	*BRCA1*	*BRCA1* (not called by WaveCNV)	*RAD51C* (G:T287A)	28	10	0.16
M	20	45	253	124	0.36	−1.16	0.18	0.29	*BRCA2* (S:K2017I)	*BRCA1/2*	N	*BRCA2* (E) K2017I Homozygous from LOH	9	25	0.36
N	117	129	367	214	−0.18	−0.41	0.43	−0.33	*BRCA1* (S:FS)	*BRCA1*	*BRCA1*	*BRCA1*-FS Homozygous from LOH	7	25	0.36
O	226	112	137	82	−0.33	−0.25	0	−0.27	None	N	N	NA	11	8	0.08
P	200	91	171	82	−0.29	−0.54	0.15	−0.40	*BRCA2* (G:FS Het->Hom), ATM(S:A538P)	N	NA	*BRCA2* –FS Homozygous ATM(S:A538P)	5	0	0.0006

Low levels of read counts in HR repair genes; CN log2 ratio using patternCNV; Mutation column indicates germline (G) or somatic (S) mutation in DNA repair pathway genes, the only deleterious mutations are the frameshift mutations (FS) and the known to be deleterious *RAD51C* mutation.

Results described by the TCGA suggest that many HGS ovarian carcinomas have both extensive changes in CN and defects in the homologous recombination (HR) pathway (6). Subsequent studies have suggested that LOH changes correlate with HR defects (35). Therefore, we summarized CN and LOH changes and examined evidence of defects in HR. The number of long LOH regions (>15 Mb), called the homologous recombination deficiency score (HRD score), in cancer genomes has been shown to correlate with the deficiencies in HR (35). We determined this HRD score using the WaveCNV package to estimate LOH and also determined an HRD-like score (counting CN decrease instead of LOH). High HRD scores (>15) were found in 10 out of the 16 low stage HGS carcinomas. The six carcinomas with low HRD scores were patients with high predicted ploidy and the ultramutated cancer for Patient D (Table 4 and Supplementary Table S5).

The majority of carcinomas with high HRD scores (HRD score ≥ 15) contained alterations in HR genes, suggesting potential defects in HR pathway (Table 4). These defects included somatic homozygous frameshift mutation in *BRCA1* (Patient N), somatic homozygous mutations in *BRCA2* (Patients F and M), known delerious (36 ) *RAD51C* mutation (Patient L), CN loss associated with *BRCA1, BRCA2* and *RAD51D* (Patient J), and CN loss associated with *BRCA1, RAD51C* and *RAD51D* (Patient I). Patient P had a germline frameshift mutation in *BRCA2* that became homozygous in the carcinoma, suggestive of a potentially deleterious mutation. Interestingly, this carcinoma has a low HRD score. In carcinomas from Patients A, G and M with high HRD scores, *BRCA1* and/or *BRCA2* mRNA were markedly downregulated by RNA-sequencing analysis (Table 4) and qRT-PCR (data not shown). Thus, it appears that decreased expression, like deleterious mutations, might contribute to genomic instability, as manifested by a high HRD score, in these low stage HGS ovarian carcinomas.

Carcinomas from three patients (Patients E, O and P), including a patient with homozygous frameshift mutation in *BRCA2* (Patient P), had relatively low HRD scores (< 10) and low genome-wide LOH burden (<0.10). The estimated CN in these carcinomas was comparable to tetraploidy (WaveCNV > 3.5, Supplementary Table S5).

To explore additional evidence for HR deficiency, we conducted hierarchical clustering of RNAseq data using a signature of genes differentially expressed between HR-deficient and HR-competent cell lines (21). Carcinomas with confirmed deleterious mutations in HR genes clustered closely with those with LOH in multiple HR genes (Supplementary Figure S2). For example, carcinomas from Patients C and G appear to cluster together and both have high HRD score, low *BRCA1* expression and *BRCA1* LOH. In addition, Patient G has a germline *BRCA2* P451Q VUS that became homozygous in the carcinoma. Carcinomas from Patients M and N cluster together and both have high HRD score and homozygous somatic mutations in *BRCA2* and *BRCA1*, respectively. Carcinomas from Patients A, B, O, E and K form another cluster with those from Patients A and B having high HRD score, the carcinoma from Patient K is just below the cutoff (HRD score < 15) and those from Patients O and E have low HRD score but have high ploidy. Carcinomas from Patients P, I and L form another group with mixed HRD score. This later group consists of Patient P with low HRD score who has a homozygous frameshift mutation in *BRCA2* and Patient I with high HRD score.

**Figure 4.**
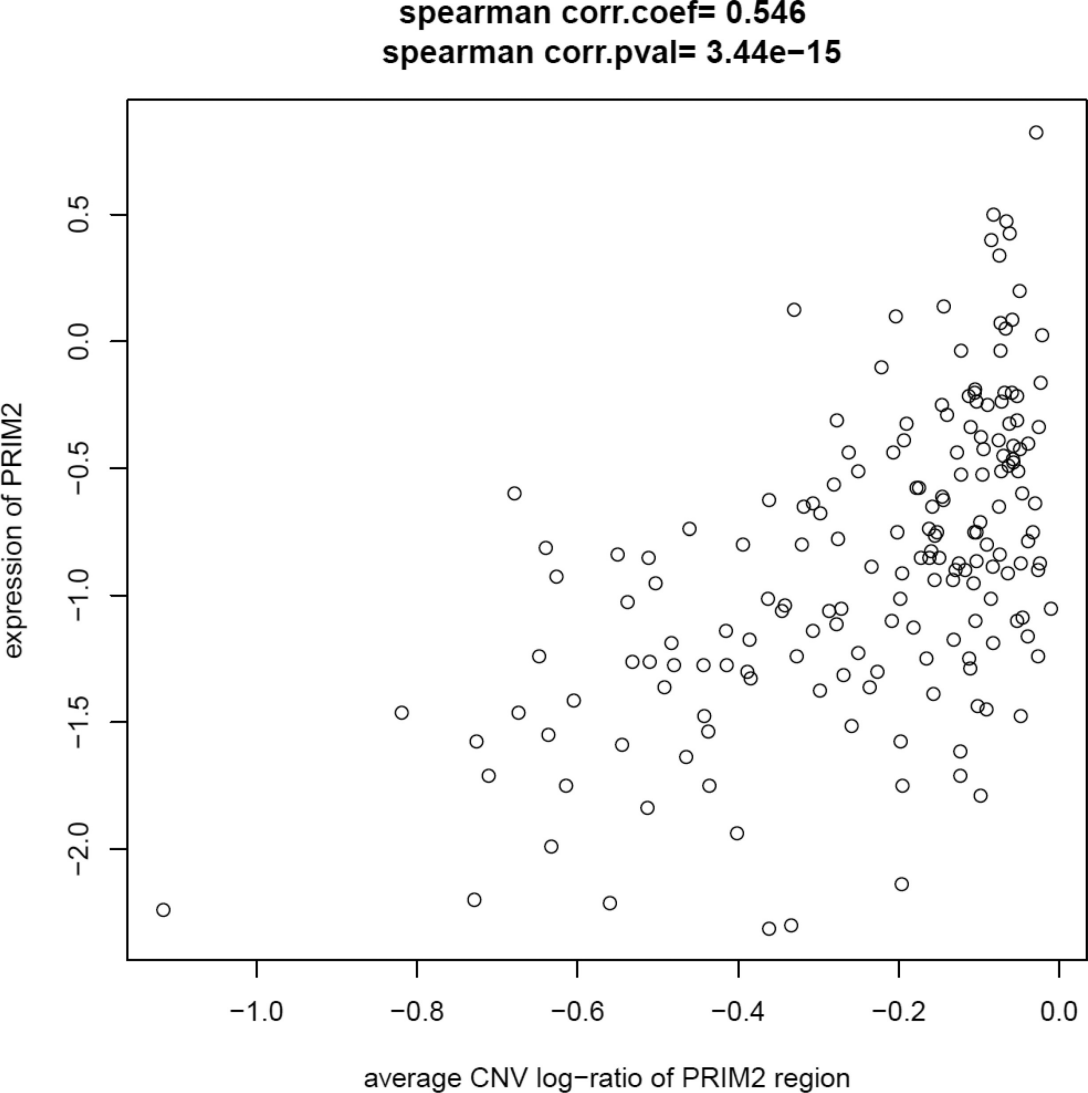
Correlation between *PRIM2* copy number and expression. Results show that somatic deletions in that region correlate with reduce expression of the *PRIM2* gene in Mayo Clinic and TCGA early stage HGS ovarian carcinomas; Spearman correlation coefficient and *P*-value shown.

Using CN data from microarrays, we identified three recurrent regions of CNA where the frequency of alteration appeared to differ between low and late stage HGS ovarian carcinomas (*TERT, SKP2* and *PRIM2*). However, only the *PRIM2* region showed a consistent CN difference between low stage and late stage cases (Supplementary Figure S4) when aCGH CN data from TCGA was used as a technical validation (*P* = 1.1 × 10^−11^). *PRIM2* also exhibited significant changes in mRNA levels with a significant expression–CN correlation (Figure 4).

The MMAF versus purity-corrected CN frequency can be used to infer the sequence of genetic alterations in the evolution of cancer (Supplementary Figure S5). In Supplementary Figure S1, we provide the MMAF versus purity-corrected CN in 15 patients. We computed the factor *F* (see Materials and Methods section) for three key genes using carcinomas with deletions with log2 ratio ≤ −0.3 and one or more somatic mutation. In Supplementary Figure S5, an *F* factor of 1 indicates that mutations arose before the CN event. Considering any somatic mutation, we found that *BRCA2* somatic mutations (chr 13) occurred before CN events with *F* = 0.98 [0.85–1.16] (*P*_wilcox_ = 0.125), based on four carcinomas. However, *TP53* (chr 17), with *F* = 0.62 [0.55–0.83] (*P*_wilcox_ = 0.01) from seven carcinomas and *BRCA1* (chr 17), with *F* = 0.63 [0.61–0.68] (*P*_wilcox_ = 0.007) from eight carcinomas, did not show the same pattern. This suggests that the accumulation of somatic mutations in *BRCA2* occurred prior to the deletion of chromosome 13 but that multiple somatic mutations occurred on chromosome 17 (*TP53* and *BRCA1*) after the CN deletion. However, in Supplementary Figure S1D, we limited to deleterious-only *TP53* mutations. In this case, we found *F* = 1.10 (1.03–1.11) with seven carcinomas, suggesting that the deleterious *TP53* mutations predate the deletion of the other functional allele. Overall, this analysis indicates that a heterozygous somatic mutation in *TP53* is an early event which, in some samples, is followed by a chromosome 17 deletion resulting in *TP53* inactivation at a relatively early point in the progression of the carcinoma. In contrast, chromosome 13 deletions are a relatively later event in the evolution of HGS ovarian cancer in this study.

Supplementary Figure S6 shows the genome-wide distribution of LOH for all samples, with the relative number of samples with an LOH event plotted in blue at the bottom of the figure. We observed that LOH was most common in chromosome 17 (14 samples, 54–70 M), followed by 4q31–35 (11 samples), 22q12 (12 samples), 9q33 (11 samples), 8p23 (10 samples), 18q (10 samples), X (10 samples), 1p (nine samples) and 13p (nine samples). Supplementary Figure S7 shows a clustered genome-wide LOH heatmap and Supplementary Figure S8 shows the LOH heatmaps for the chromosomes with the greatest LOH. Despite the widespread LOH in most samples, carcinomas from Patients E, O and P, which had high mean ploidy, had low LOH as indicated above.

Finally, to identify somatic structural variation (SVs) such as large INDELs and interchromosomal translocations, we applied the CREST bioinformatics tool and characterized somatic SVs uncovered in these samples. Results shown in Supplementary Table S9 indicates heterogeneity in SV burden in these tumors. Interestingly, the ultramutated carcinoma from Patient D has no detectable SV in addition to little CNV. Genes affected by recurrent translocation in at least two samples are shown in Supplementary Table S10. DAVID Bioinformatics Analysis (37) of functional annotation of genes affected by recurrent SVs in these samples indicate cell projection, morphogenesis and GTPase regulator activities as biological functions affected by these genes (Supplementary Table S11). It is striking that 43% of the SV events (Supplementary Table S12) cluster in groups of SVs (separated by 1 MB or less). The paucity of Kataegis events but presence of clustered SVs in these samples raises the possibility that these might be a preferential outcome of showers of DNA damage in OCs with repair defects.

## DISCUSSION

Using a rare set of low stage HGS ovarian carcinomas from the Mayo Clinic, we report the first whole genome sequencing in ovarian cancer and identify early mutational events in this disease. Integrated analysis of whole genome sequences, RNA sequences and CN changes produced five major findings: (i) frequent mutations and LOH in *TP53*, (2) *PRIM2* loss as a frequent genetic alteration, (iii) frequent HR defects and genome-wide CNA ,(iv) clustering of structural variants in genome, and (v) polyploidy in certain low stage HGS ovarian cancers, .

### TP53 mutations and LOH in low stage HGS ovarian cancer

Our analysis indicates that *TP53* is the most frequently mutated gene in low stage HGS ovarian cancer. This observation, taken together with previous studies indicating that *TP53* mutations are ubiquitous in late stage HGS ovarian cancer (6,33), highlights *TP53* mutations as the pathognomonic feature of HGS ovarian cancer. As this patient collection consisted of low stage HGS carcinomas, this finding lends further support to prior studies of early/low stage HGS, which showed somatic *TP53* mutations in serous tubal intraepithelial carcinomas from women with germline mutations in *BRCA1* or *BRCA2* (38). Our results also indicate that deleterious *TP53* mutations precede CN deletion events that eliminated the wild type copy of *TP53*. Collectively, these results suggest that the most frequent sequence of events leading to tumorigenesis in HGS ovarian cancer is an early somatic mutation in *TP53* followed by genome-wide LOH that often includes CN loss in chromosomes 13 or 17.

Detailed analysis of *TP53* mutations in our study led to three additional observations. First, we identified loss of the wild type copy via deletion or LOH in 10 out of 16 low stage HGS carcinomas with mutant *TP53*. Second, in the carcinomas without allelic loss of wild type *TP53*, expression analysis showed inactivation of the transcriptional activity of the wild type copy in our RNA sequence data for four more carcinomas. Third, one cancer without any *TP53* mutation (Patient H) had the smallest number of deleterious somatic mutations (four missense, one nonsense and one splice-site mutations), yet had a relatively high HRD score. Interestingly, this carcinoma also had no mutations in the high frequency driver genes found by TCGA, namely, *NF1, CDK12, FAT3, GAGRA6, BRCA1* and *RB1*. It did, however, have a somatic *CHEK2* mutation, which might well account for the genomic instability as manifested by the relatively high HRD score. Interestingly, Patient H is also the only patient with stage I ovarian carcinoma who experienced recurrence and died from the disease (Supplementary Tables S1 and Table S13).

In this series of low stage HGS ovarian carcinomas, we observed four *TP53* mutations (R273C, R248W, R248Q and R175H) that were previously described as ‘neomorphic’ (gain of function, GOF) changes (39). The frequency of neomorphic mutations in the Mayo Clinic carcinomas was similar to that reported in the TCGA set of primarily late stage carcinomas (*P*-value 1.0, *t*-test). Some of these neomorphic mutations have been shown to produce metastatic tumors in mouse model systems. For example, R172H (equivalent to R175H in human) mutant mice produce highly metastatic tumors (40,41). It is therefore interesting that a neomorphic *TP53* mutation, R175H, is found in early stage ovarian cancer (Patient C) with stage IB at diagnosis. It is possible that incidental finding of some of these early stage carcinomas may have limited the metastatic potential. In addition, the metastatic behavior of R172H mutant in mice is also host- and dose-dependent (40,41), and host factors may have limited the metastatic potential of R175H in this particular case of HGS ovarian carcinoma. Consistent with the role of GOF *TP53* mutants in promoting metastatic potential, Kang *et al*. observed that patients with GOF *TP53* mutations in the TCGA cohort were more likely to develop distant metastasis (42). However, no significant association between GOF *TP53* mutations and clinical outcome was observed (42). These results are also consistent with prior studies by Ahmed *et al.*, who also found no significant association between two types of *TP53* mutations (missense versus non-missense) and clinical outcome (33).

The other three patients with putative neomorphic *TP53* mutations were diagnosed with stage II carcinomas. Two of them (Patients K and O) had low HRD scores and no defects in HR genes. Since HR deficiency is associated with better response to platinum-based chemotherapy and PARP inhibitors (6,43–44), it would be important to investigate the extent to which some of the neomorphic *TP53* mutations are associated with HR proficiency and whether these mutants are associated with poor response to platinum-based chemotherapy or PARP inhibitors.

### PRIM2 loss as a frequent alteration in early/low stage HGS ovarian cancer

Our results showed that the *PRIM2* gene was more frequently deleted in early stage than in late stage HGS ovarian cancer. *PRIM2* encodes DNA primase polypeptide 2, which is involved in DNA replication and synthesizes Okazaki fragments to seed DNA replication starting points. It is possible that lower levels of this enzyme facilitate structural rearrangements by disruption of DNA replication. Studies in the *Saccharomyces cerevisiae* model system indicate that defects in lagging-strand replication are repaired by a *RAD51*-dependent mechanism (45). Therefore, both *PRIM2* loss and *RAD51* downregulation in HGS cancer may result in utilization of the error-prone repair pathway (e.g. non-homologous end joining) and may contribute to the extensive rearrangements found in HGS ovarian cancer. Interestingly, both *PRIM2* loss and *CHEK2* mutation are observed in the cancer from Patient H without any *TP53* mutations. The CNAs observed this cancer are comparable to other early stage HGS ovarian carcinomas with *TP53* mutations and HR defects, suggesting the possibility that *PRIM2* and *CHEK2* loss in Patient H may contribute to comparable levels of structural alterations.

### HR defects and genome-wide LOH

The pattern of LOH found in early stage HGS ovarian carcinomas is consistent with the pattern previously reported in late stage disease (19). Wang *et al.* reported chromosome 17 and 13 as the two chromosomes most frequently exhibiting large-scale LOH. While our results replicate the earlier findings for chromosome 17 (LOH in 14 samples), chromosome 13 LOH occurred in only nine samples, and other regions were more frequently subject to LOH [22q12 (12 samples), 4q31–35 (11 samples), 9q33 (11 samples), 8p23 (10 samples), 18q (10 samples) and X (10 samples)]. This lower frequency of chromosome 13 LOH compared to chromosome 17 reinforces our thesis that chromosome 13 LOH may be a later event. Although this conclusion is limited by small sample size, this is consistent with our joint analysis of mutations and deletions on chromosome 13, which showed deletions to be late events.

### SVs and clustered damage

In contrast to a number of cancers, where clusters of point mutations called Kataegis events are observed, few such events were observed in the low stage HGS ovarian cancers examined here. On the other hand, many of the SVs detected in the low stage ovarian cancer samples occurred in clusters. These results suggest that DNA damage might have occurred in clusters in these tumors but, perhaps because of differences in repair capacities or anomalous function of the topo-isomerase genes, led to clusters of double-strand breaks and subsequent SVs in early ovarian cancer with lower rates of Kataegis events than observed in other cancers (46).

### Polyploidy in carcinomas with minimal LOH

The Mayo Clinic carcinomas with high HRD scores showed LOH alteration, consistent with Wang's ‘Cluster HiA’ (High LOH) carcinomas with HR defects and good response to platinum therapy (19). The Mayo Clinic carcinomas with low levels of LOH had low HRD scores and were similar to Wang's cluster Lo carcinomas, which were less responsive to platinum therapies (19), presumably due to a functional HR pathway. Our observations also indicate that carcinomas with low LOH are associated with polyploidy, which has also been implicated in cisplatin resistance (47) and associated with worse prognosis (48).

We are the first to show the co-existence of polyploidy and low genomic LOH in HGS ovarian carcinoma. Co-existence of polyploidy and functional HR in these samples would be consistent with prior studies in yeast indicating the synthetic lethality of tetraploidy and HR defects in yeast (49). However, there is other evidence that tetraploid tumors can have defective HR (via *BRCA1* mutation) at the same rate as diploid tumors (50). It is also possible that the tetraploid tumors are not able to display a high HRD score despite the presence of a HR defect.

Previous studies in mice identified a possible mechanism of ovarian epithelial tumorigenesis via a transition to a tetraploid state (51) followed by aneuploidy changes as long as *TP53* mutations were present. Carcinomas from Patients O, P and E may be in the earliest stages of this transformation and have not yet undergone transition toward the diploid state with a high frequency of LOH despite strong evidence of HR inactivation in Patient P (homozygous frameshift mutation of *BRCA2*). Thus, some of those tetraploid tumors may still have a defective HR pathway despite exhibiting a low HRD score. Nevertheless many tetraploid tumors may still be HR competent to explain the higher recurrence rate of low LOH tumors in (19). This hypothesis is also supported by the clustering of carcinomas from Patients O, P and E with other samples with high HRD scores based on the gene expression signature previously described (21).

Lastly, we found one ultramutated carcinoma (Patient D), which initially appeared completely different from other patients. This cancer had *POLE* V411L mutation that was characterized as one of the hotspot mutations in ultramutated endometrial and colorectal carcinomas (25,52). This patient had minimal levels of SV, CNV and LOH and thus the same pattern as seen in other carcinomas with high rate of somatic SNVs (53). For this cancer, it is likely that*POLE* mutation contributes to an ultramutated phenotype with low levels of structural alterations.

## CONCLUSIONS

Although our sample size is limited due to the rarity of low stage HGS ovarian carcinomas, the overall genomic landscape of these carcinomas is remarkably similar to late stage HGS ovarian carcinomas in mutation spectrum, burden and structural rearrangement. These results, which are consistent with the earlier observation by Kobel *et al*. that early and late stage HGS carcinomas are indistinguishable based on immunohistochemical analysis 21 biomarkers (54), extend these findings to the whole-genome level.

While *TP53* mutation is a key early event, we have not identified an event that would lead to a high rate of somatic mutations to initially inactivate *TP53*. Additional studies evaluating candidate mechanisms, such as *APOBEC3B* (46) are needed to understand initial carcinogenic events that lead to inactivation of *TP53*. Our data, which show that mutations in *TP53* may be driver events in ovarian carcinogenesis, provide additional impetus for efforts to restore the function of *TP53* or exploit its deficit in patients with HGS ovarian cancer. The present results also indicate that chromosome 13 events (CN changes or LOH) are later than chromosome 17 events. We have also identified one early stage HGS ovarian cancer with an ultramutated phenotype, but LOH analysis has revealed that this ultramutated phenotype is superimposed on a background of low-levels CN and LOH events consistent with many of the other low stage, HGS ovarian carcinomas, indicating that this is also a late event. In addition, we identified several carcinomas with low LOH and high estimated ploidy which is consistent with the model in which some HGS ovarian cancers are initially tetraploid and become nearly diploid due to LOH.

Despite heterogeneity in somatic sequence variations, CNAs and structural variations, none of these somatic genotypes appears to be associated with clinical outcome. Only stage appears to be strongly linked to patient survival, as six of eight patients with stage I HGS ovarian carcinomas are still alive (median follow-up 101 months), whereas only two of eight with stage 2 disease are still alive (median follow-up 79 months). Collectively, these observations provide new insight into the biology and likely pathogenesis of early stage HGS ovarian cancer.

## SUPPLEMENTARY DATA

Supplementary Data are available at NAR Online.

SUPPLEMENTARY DATA

## References

[1] Jemal A., Bray F., Center M.M., Ferlay J., Ward E., Forman D. (2011). Global cancer statistics. CA Cancer J. Clin..

[2] Bell D.A. (2005). Origins and molecular pathology of ovarian cancer. Mod. Pathol..

[3] Shih Ie M., Kurman R.J. (2004). Ovarian tumorigenesis: a proposed model based on morphological and molecular genetic analysis. Am. J. Pathol..

[4] Chan J.K., Cheung M.K., Husain A., Teng N.N., West D., Whittemore A.S., Berek J.S., Osann K. (2006). Patterns and progress in ovarian cancer over 14 years. Obstet. Gynecol..

[5] Seidman J.D., Chauhan S. (2002). Evaluation of the relationship between adenosarcoma and carcinosarcoma and a hypothesis of the histogenesis of uterine sarcomas. Int. J. Gynecol. Pathol..

[6] Cancer Genome Atlas Research Network (2011). Integrated genomic analyses of ovarian carcinoma. Nature.

[7] Li H., Durbin R. (2009). Fast and accurate short read alignment with Burrows-Wheeler Transform. Bioinformatics.

[8] DePristo M.A., Banks E., Poplin R., Garimella K.V., Maguire J.R., Hartl C., Philippakis A.A., del Angel G., Rivas M.A., Hanna M. (2011). A framework for variation discovery and genotyping using next-generation DNA sequencing data. Nat. Genet..

[9] Larson D.E., Harris C.C., Chen K., Koboldt D.C., Abbott T.E., Dooling D.J., Ley T.J., Mardis E.R., Wilson R.K., Ding L. (2012). SomaticSniper: identification of somatic point mutations in whole genome sequencing data. Bioinformatics.

[10] Asmann Y.W., Middha S., Hossain A., Baheti S., Li Y., Chai H.S., Sun Z., Duffy P.H., Hadad A.A., Nair A. (2012). TREAT: a bioinformatics tool for variant annotations and visualizations in targeted and exome sequencing data. Bioinformatics.

[11] Dees N.D., Zhang Q., Kandoth C., Wendl M.C., Schierding W., Koboldt D.C., Mooney T.B., Callaway M.B., Dooling D., Mardis E.R. (2012). MuSiC: identifying mutational significance in cancer genomes. Genome Res..

[12] Trapnell C., Pachter L., Salzberg S.L. (2009). TopHat: discovering splice junctions with RNA-Seq. Bioinformatics.

[13] Anders S., Pyl P.T., Huber W. (2014). HTSeq-a Python framework to work with high-throughput sequencing data. Bioinformatics.

[14] Horaitis O., Talbot C.C. Jr, Phommarinh M., Phillips K.M., Cotton R.G. (2007). A database of locus-specific databases. Nat. Genet..

[15] Ota T., Clayton A.C., Minot D.M., Shridhar V., Hartmann L.C., Gilks C.B., Chien J.R. (2011). Minichromosome maintenance protein 7 as a potential prognostic factor for progression-free survival in high-grade serous carcinomas of the ovary. Mod. Pathol..

[16] Wang C., Evans J.M., Bhagwate A.V., Prodduturi N., Sarangi V., Middha M., Sicotte H., Vedell P.T., Hart S.N., Oliver G.R. (2014). PatternCNV: a versatile tool for detecting copy number changes from exome sequencing data. Bioinformatics.

[17] Sun W., Wright F.A., Tang Z., Nordgard S.H., Loo P.V., Yu T., Kristensen V.N., Perou C.M. (2009). Integrated study of copy number states and genotype calls using high-density SNP arrays. Nucleic Acids Res..

[18] Holt C., Losic B., Pai D., Zhao Z., Trinh Q., Syam S., Arshadi N., Jang G.H., Ali J., Beck T. (2014). WaveCNV: allele-specific copy number alterations in primary tumors and xenograft models from next-generation sequencing. Bioinformatics.

[19] Wang Z.C., Birkbak N.J., Culhane A.C., Drapkin R., Fatima A., Tian R., Schwede M., Alsop K., Daniels K.E., Piao H. (2012). Profiles of genomic instability in high-grade serous ovarian cancer predict treatment outcome. Clin. Cancer Res..

[20] Skirnisdottir I., Mayrhofer M., Rydaker M., Akerud H., Isaksson A. (2012). Loss-of-heterozygosity on chromosome 19q in early-stage serous ovarian cancer is associated with recurrent disease. BMC Cancer.

[21] Peng G., Chun-Jen Lin C., Mo W., Dai H., Park Y.Y., Kim S.M., Peng Y., Mo Q., Siwko S., Hu R. (2014). Genome-wide transcriptome profiling of homologous recombination DNA repair. Nat. Commun..

[22] Wang J., Mullighan C.G., Easton J., Roberts S., Heatley S.L., Ma J., Rusch M.C., Chen K., Harris C.C., Ding L. (2011). CREST maps somatic structural variation in cancer genomes with base-pair resolution. Nat. Methods.

[23] Kandoth C., Schultz N., Cherniack A.D., Akbani R., Liu Y., Shen H., Robertson A.G., Pashtan I., Shen R., Benz C.C. (2013). Integrated genomic characterization of endometrial carcinoma. Nature.

[24] Church D.N., Briggs S.E., Palles C., Domingo E., Kearsey S.J., Grimes J.M., Gorman M., Martin L., Howarth K.M., Hodgson S.V. (2013). DNA polymerase epsilon and delta exonuclease domain mutations in endometrial cancer. Hum. Mol. Genet..

[25] Cancer Genome Atlas Network (2012). Comprehensive molecular characterization of human colon and rectal cancer. Nature.

[26] Jones S., Wang T.-L., Shih I.-M., Mao T.-L., Nakayama K., Roden R., Glas R., Slamon D., Diaz L.A., Vogelstein B. (2010). Frequent mutations of chromatin remodeling gene ARID1A in ovarian clear cell carcinoma. Science.

[27] Lawrence M.S., Stojanov P., Polak P., Kryukov G.V., Cibulskis K., Sivachenko A., Carter S.L., Stewart C., Mermel C.H., Roberts S.A. (2013). Mutational heterogeneity in cancer and the search for new cancer-associated genes. Nature.

[28] Laird P.W., Jaenisch R. (1994). DNA methylation and cancer. Hum. Mol. Genet..

[29] Alexandrov L.B., Nik-Zainal S., Wedge D.C., Aparicio S.A., Behjati S., Biankin A.V., Bignell G.R., Bolli N., Borg A., Borresen-Dale A.L. (2013). Signatures of mutational processes in human cancer. Nature.

[30] Thorvaldsdottir H., Robinson J.T., Mesirov J.P. (2013). Integrative genomics viewer (IGV): high-performance genomics data visualization and exploration. Brief. Bioinform..

[31] Robinson J.T., Thorvaldsdottir H., Winckler W., Guttman M., Lander E.S., Getz G., Mesirov J.P. (2011). Integrative genomics viewer. Nat. Biotechnol..

[32] Vogelstein B., Kinzler K.W. (2004). Cancer genes and the pathways they control. Nat. Med..

[33] Ahmed A.A., Etemadmoghadam D., Temple J., Lynch A.G., Riad M., Sharma R., Stewart C., Fereday S., Caldas C., Defazio A. (2010). Driver mutations in TP53 are ubiquitous in high grade serous carcinoma of the ovary. J. Pathol..

[34] Kindelberger D.W., Lee Y., Miron A., Hirsch M.S., Feltmate C., Medeiros F., Callahan M.J., Garner E.O., Gordon R.W., Birch C. (2007). Intraepithelial carcinoma of the fimbria and pelvic serous carcinoma: evidence for a causal relationship. Am. J. Surg. Pathol..

[35] Abkevich V., Timms K.M., Hennessy B.T., Potter J., Carey M.S., Meyer L.A., Smith-McCune K., Broaddus R., Lu K.H., Chen J. (2012). Patterns of genomic loss of heterozygosity predict homologous recombination repair defects in epithelial ovarian cancer. Br. J. Cancer.

[36] Somyajit K., Subramanya S., Nagaraju G. (2012). Distinct roles of FANCO/RAD51C protein in DNA damage signaling and repair: implications for Fanconi anemia and breast cancer susceptibility. J. Biol. Chem..

[37] Huang D.W., Sherman B.T., Tan Q., Collins J.R., Alvord W.G., Roayaei J., Stephens R., Baseler M.W., Lane H.C., Lempicki R.A. (2007). The DAVID Gene Functional Classification Tool: a novel biological module-centric algorithm to functionally analyze large gene lists. Genome Biol..

[38] Crum C.P., Drapkin R., Kindelberger D., Medeiros F., Miron A., Lee Y. (2007). Lessons from BRCA: the tubal fimbria emerges as an origin for pelvic serous cancer. Clin. Med. Res..

[39] Muller P.A., Vousden K.H. (2014). Mutant p53 in cancer: new functions and therapeutic opportunities. Cancer Cell.

[40] Lang G.A., Iwakuma T., Suh Y.A., Liu G., Rao V.A., Parant J.M., Valentin-Vega Y.A., Terzian T., Caldwell L.C., Strong L.C. (2004). Gain of function of a p53 hot spot mutation in a mouse model of Li-Fraumeni syndrome. Cell.

[41] Olive K.P., Tuveson D.A., Ruhe Z.C., Yin B., Willis N.A., Bronson R.T., Crowley D., Jacks T. (2004). Mutant p53 gain of function in two mouse models of Li-Fraumeni syndrome. Cell.

[42] Kang H.J., Chun S.M., Kim K.R., Sohn I., Sung C.O. (2013). Clinical relevance of gain-of-function mutations of p53 in high-grade serous ovarian carcinoma. PloS One.

[43] Bryant H.E., Schultz N., Thomas H.D., Parker K.M., Flower D., Lopez E., Kyle S., Meuth M., Curtin N.J., Helleday T. (2005). Specific killing of BRCA2-deficient tumours with inhibitors of poly(ADP-ribose) polymerase. Nature.

[44] Farmer H., McCabe N., Lord C.J., Tutt A.N., Johnson D.A., Richardson T.B., Santarosa M., Dillon K.J., Hickson I., Knights C. (2005). Targeting the DNA repair defect in BRCA mutant cells as a therapeutic strategy. Nature.

[45] Li H., Durbin R. (2009). Fast and accurate short read alignment with Burrows–Wheeler transform. Bioinformatics.

[46] Leonard B., Hart S.N., Burns M.B., Carpenter M.A., Temiz N.A., Rathore A., Vogel R.I., Nikas J.B., Law E.K., Brown W.L. (2013). APOBEC3B upregulation and genomic mutation patterns in serous ovarian carcinoma. Cancer Res..

[47] Davis E., Teng H., Bilican B., Parker M.I., Liu B., Carriera S., Goding C.R., Prince S. (2008). Ectopic Tbx2 expression results in polyploidy and cisplatin resistance. Oncogene.

[48] Lassus H., Staff S., Leminen A., Isola J., Butzow R. (2011). Aurora-A overexpression and aneuploidy predict poor outcome in serous ovarian carcinoma. Gynecol. Oncol..

[49] Storchova Z., Breneman A., Cande J., Dunn J., Burbank K., O'Toole E., Pellman D. (2006). Genome-wide genetic analysis of polyploidy in yeast. Nature.

[50] Pradhan M., Risberg B.A., Trope C.G., van de Rijn M., Gilks C.B., Lee C.H. (2010). Gross genomic alterations and gene expression profiles of high- grade serous carcinoma of the ovary with and without BRCA1 inactivation. BMC Cancer.

[51] Lv L., Zhang T., Yi Q., Huang Y., Wang Z., Hou H., Zhang H., Zheng W., Hao Q., Guo Z. (2012). Tetraploid cells from cytokinesis failure induce aneuploidy and spontaneous transformation of mouse ovarian surface epithelial cells. Cell Cycle.

[52] Kandoth C., Schultz N., Cherniack A.D., Akbani R., Liu Y., Shen H., Robertson A.G., Pashtan I., Shen R., Benz C.C., Cancer Genome Atlas Research Network (2013). Integrated genomic characterization of endometrial carcinoma. Nature.

[53] Ciriello G., Miller M.L., Aksoy B.A., Senbabaoglu Y., Schultz N., Sander C. (2013). Emerging landscape of oncogenic signatures across human cancers. Nat. Genet..

[54] Kobel M., Kalloger S.E., Boyd N., McKinney S., Mehl E., Palmer C., Leung S., Bowen N.J., Ionescu D.N., Rajput A. (2008). Ovarian carcinoma subtypes are different diseases: implications for biomarker studies. PLoS Med..

