# Supplementary material for: TP53 mutations, tetraploidy and homologous recombination repair defects in early stage high-grade serous ovarian cancer
Source: Nucleic Acids Res. 2015 Apr 27;43(14):6945–58. doi: 10.1093/nar/gkv111 (PMC4538798; doi:10.1093/nar/gkv111)
Supplement: SUPPLEMENTARY DATA [file supp_43_14_6945__index.html]

TP53 mutations, tetraploidy and homologous recombination repair defects in early stage high-grade serous ovarian cancer — TP53 mutations, tetraploidy and homologous recombination repair defects in early stage high-grade serous ovarian cancer — TP53 mutations, tetraploidy and homologous recombination repair defects in early stage high-grade serous ovarian cancer — TP53 mutations, tetraploidy and homologous recombination repair defects in early stage high-grade serous ovarian cancer — SUPPLEMENTARY DATA 

# *TP53* mutations, tetraploidy and homologous recombination repair defects in early stage high-grade serous ovarian cancer

## SUPPLEMENTARY DATA

**Files in this Data Supplement:**

- SUPPLEMENTARY DATA
- SUPPLEMENTARY DATA
- SUPPLEMENTARY DATA
