# Supplementary material for: TP53 mutations, tetraploidy and homologous recombination repair defects in early stage high-grade serous ovarian cancer
Source: Nucleic Acids Res. 2015 Apr 27;43(14):6945–58. doi: 10.1093/nar/gkv111 (PMC4538798; doi:10.1093/nar/gkv111)
Supplement: SUPPLEMENTARY DATA [file supp_gkv111_nar-03083-x-2014-File006.pdf]

Figure S1. Mean somatic mutation allele frequency for any somatic variants in a 1MB window centered on key genes as a function of purity corrected copy number levels.

A

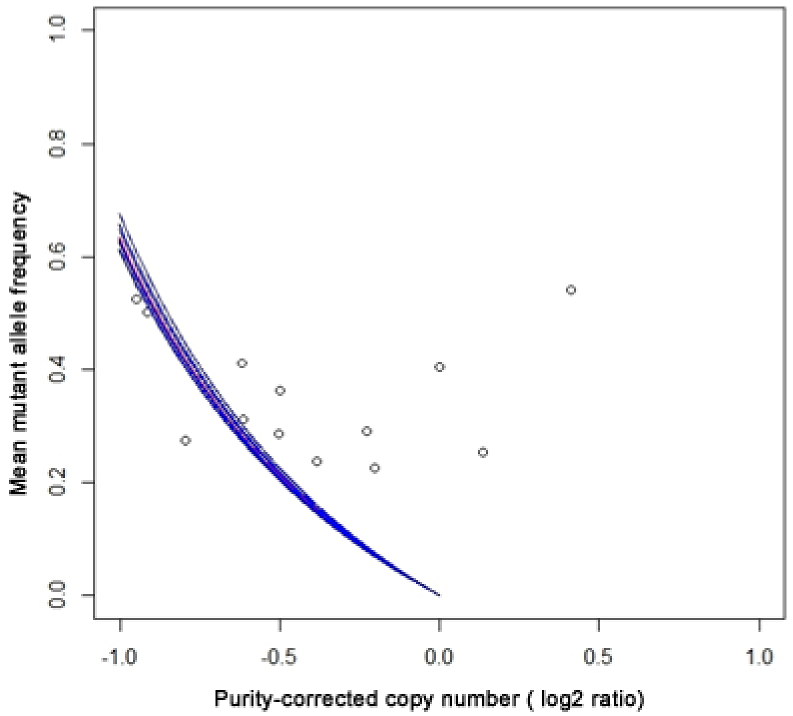

B

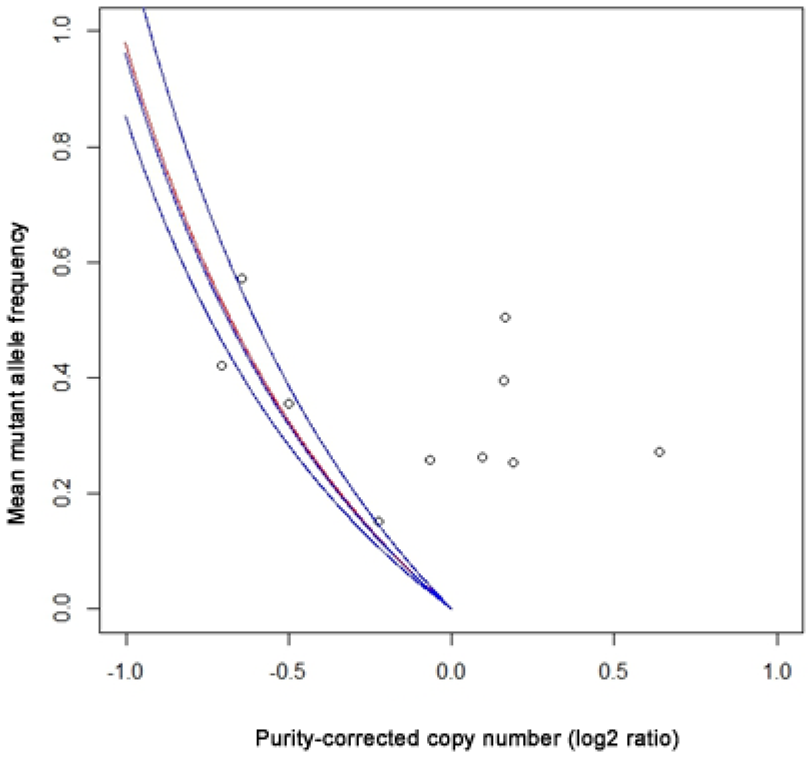

C

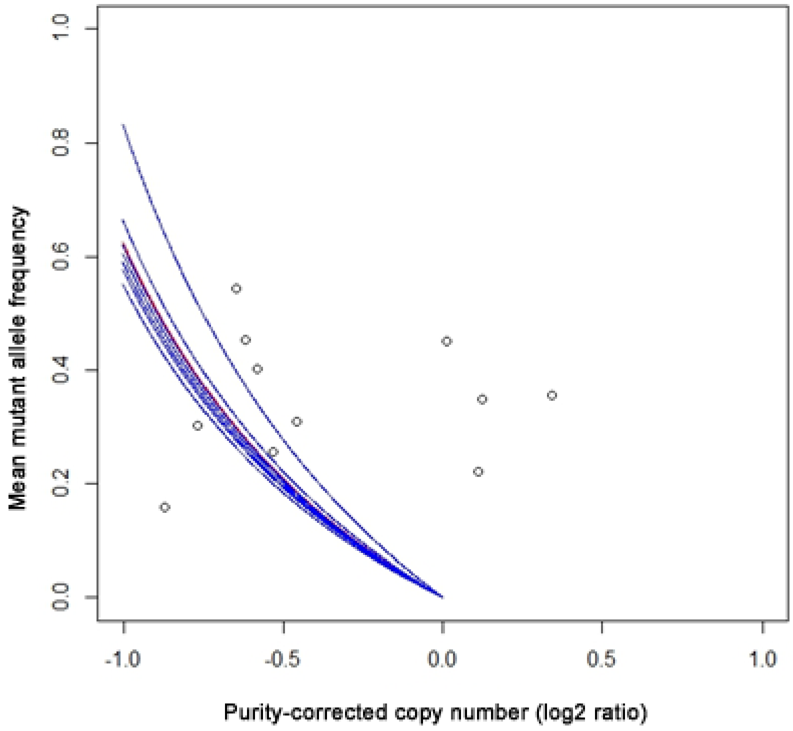

Mean mutant allele frequency
Purity-corrected copy number (log2 ratio)
0.0
0.2
0.4
0.6
0.8
1.0
-1.0
-0.5
0.0
0.5
1.0

D

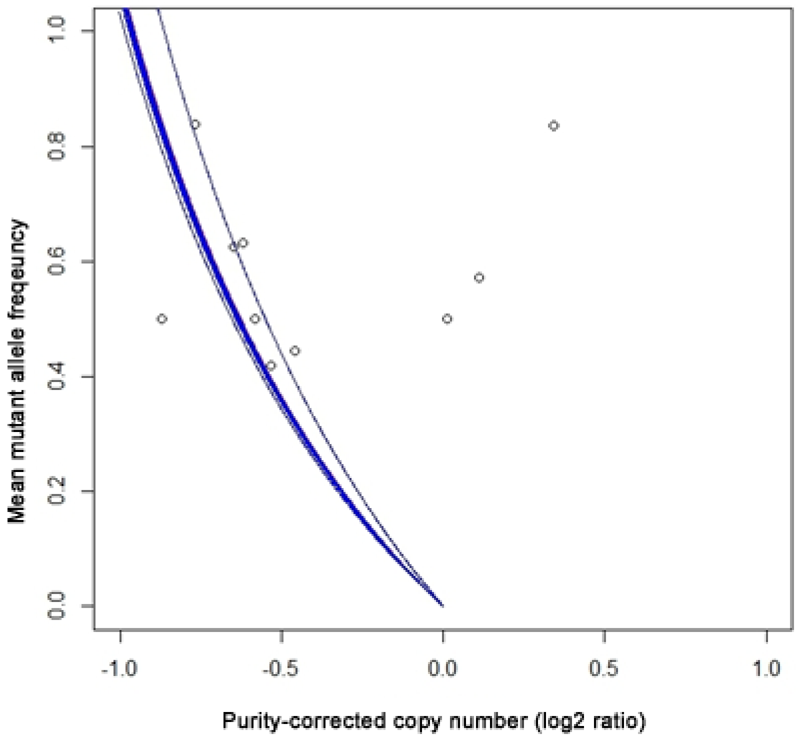

Mean mutant allele freqeuncy
Purity-corrected copy number (log2 ratio)
0.0
0.2
0.4
0.6
0.8
1.0
-1.0
-0.5
0.0
0.5
1.0

The fact that the deletion level is not at -1 reflects the fraction of tumor clones carrying the deletion. The mean mutant allele frequency also depends on wether the mutation predates the acquisition of a deletion by a sample. The curves in red denotes the fit of Equation 2 to the mutations in samples with deletion copy number, and the blue lines are fit using leave-one-out cross-validation subsets. (A) *BRCA1* (F=0.63 ,p=0.007), (B) *BRCA2* (F=0.98, p=0.125), (C) *TP53* (F=0.62, p=0.01), (D) *TP53* deleterious mutations only (F=1.10).

Figure S2. Differentially expressed gene signature for HR defect.

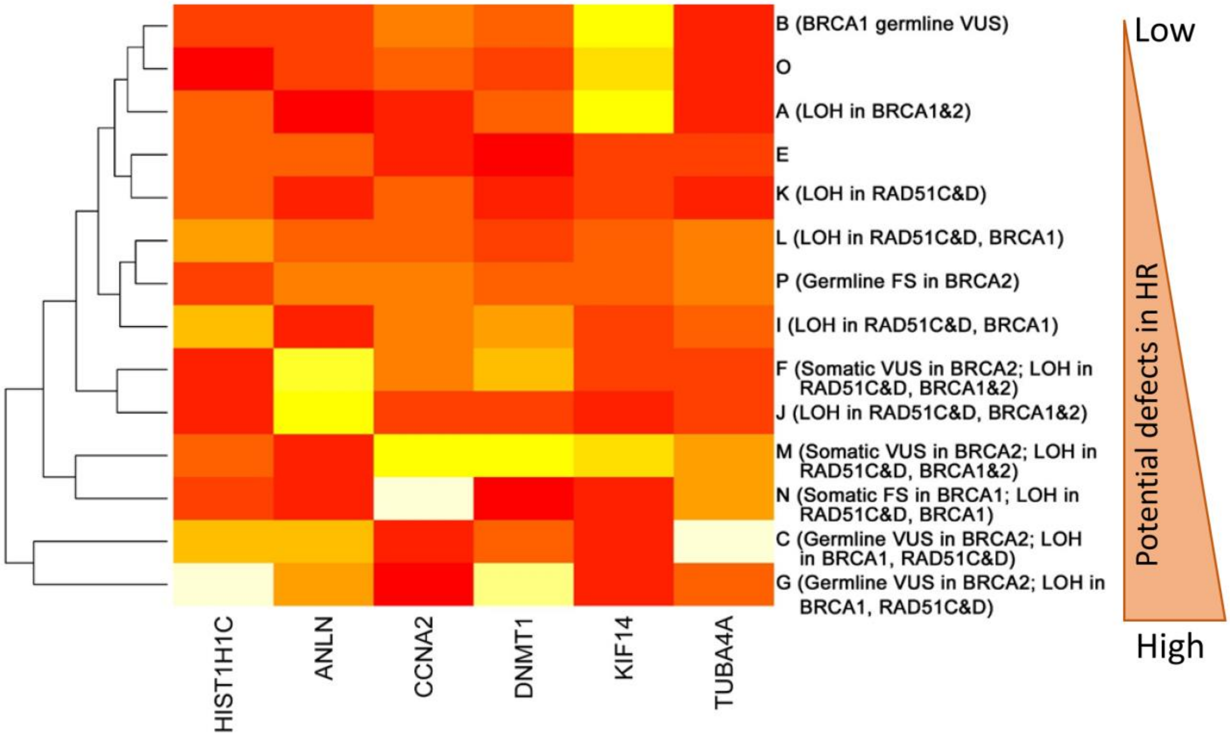

Genes differentially expressed between HR deficient cell lines and HR competent cells lines (Peng et al., 2014). Since the signature was derived from gene expression microarrays, Panel a only included genes in the top fifty percent of variance/median ratio in sequencing and expected log2ratio of |log2|>0.5 between the two categories.

Figure S3. P53 immunohistochemistry.

A.

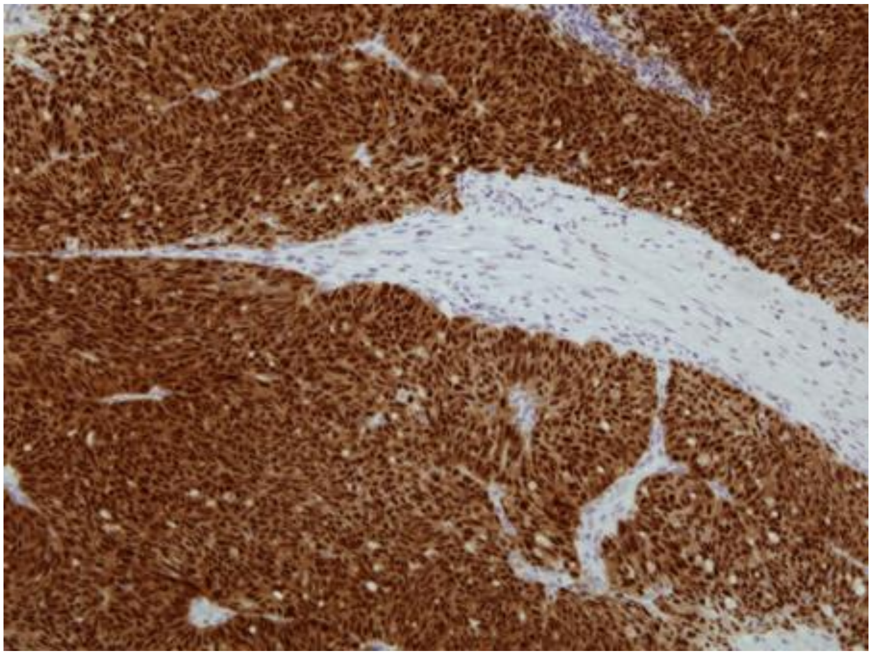

B.

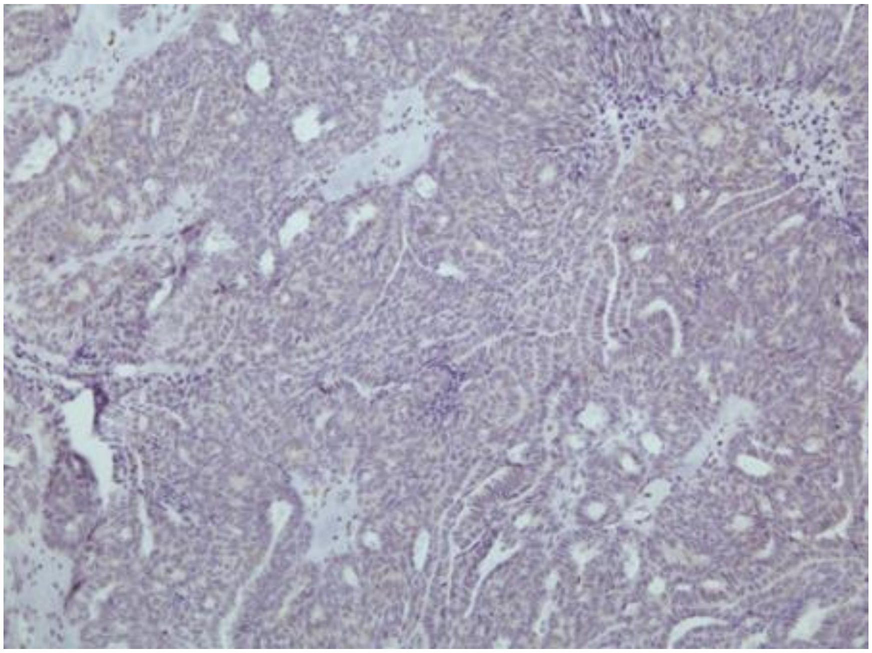

(A) Representative immunohistochemical staining pattern of p53 in tumors with p53 mutations. Minimal staining of p53 is observed in stroma. (B) Tumor sample that is negative for p53 staining.

Figure S4. Technical validation of copy number in aCGH.

Comparison of aCGH results for early versus late TCGA, as technical validation of TCGA focal amplification/deletions that has significant differences between the early stage HGS (Mayo or TCGA as determined by SNP arrays) and the TCGA late stage HGS samples. The most significant region, containing the *PRIM2* gene, has increased frequency of copy number loss in the late stage samples.

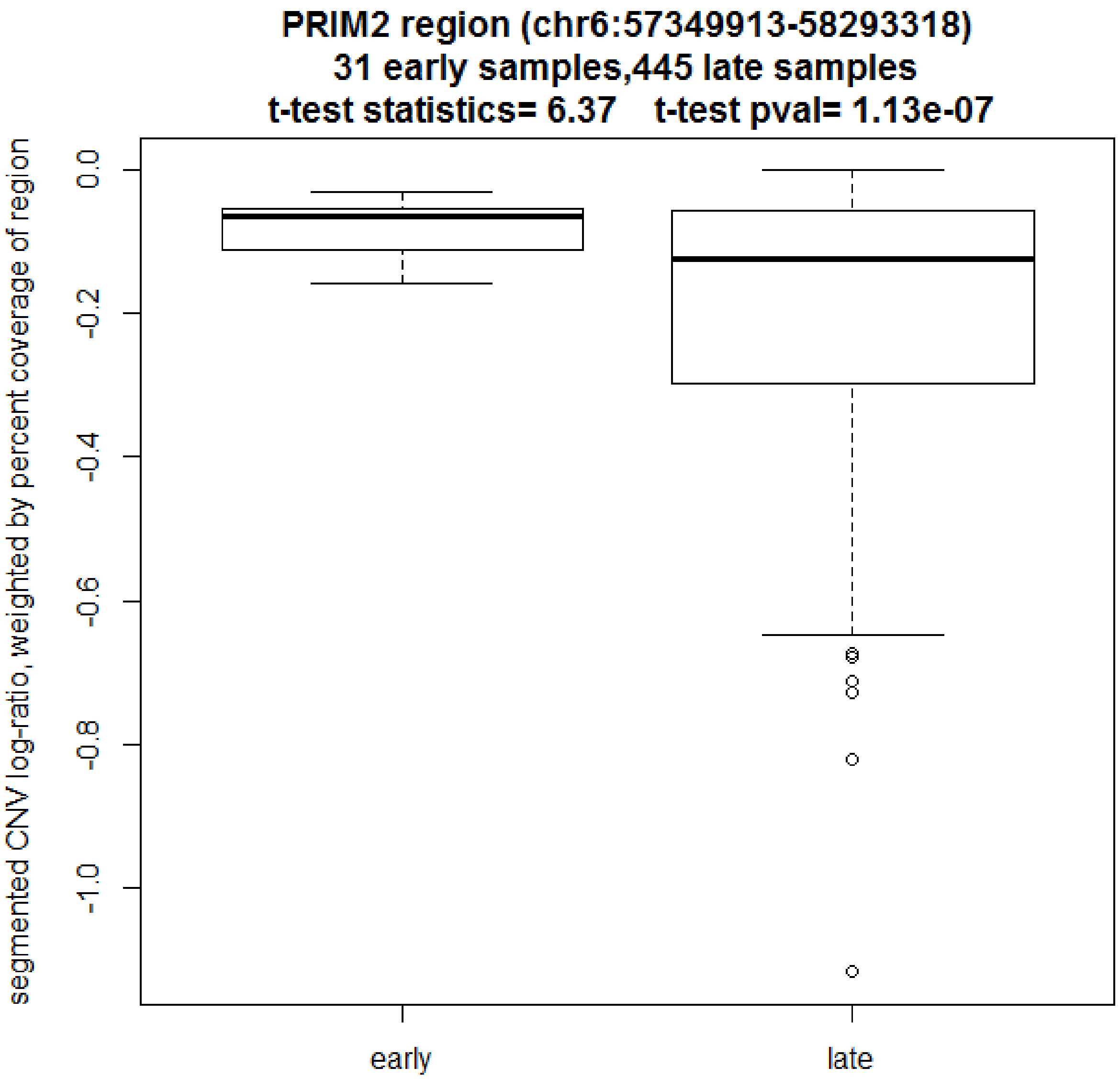

Figure S5. Schematic of tumor evolution.

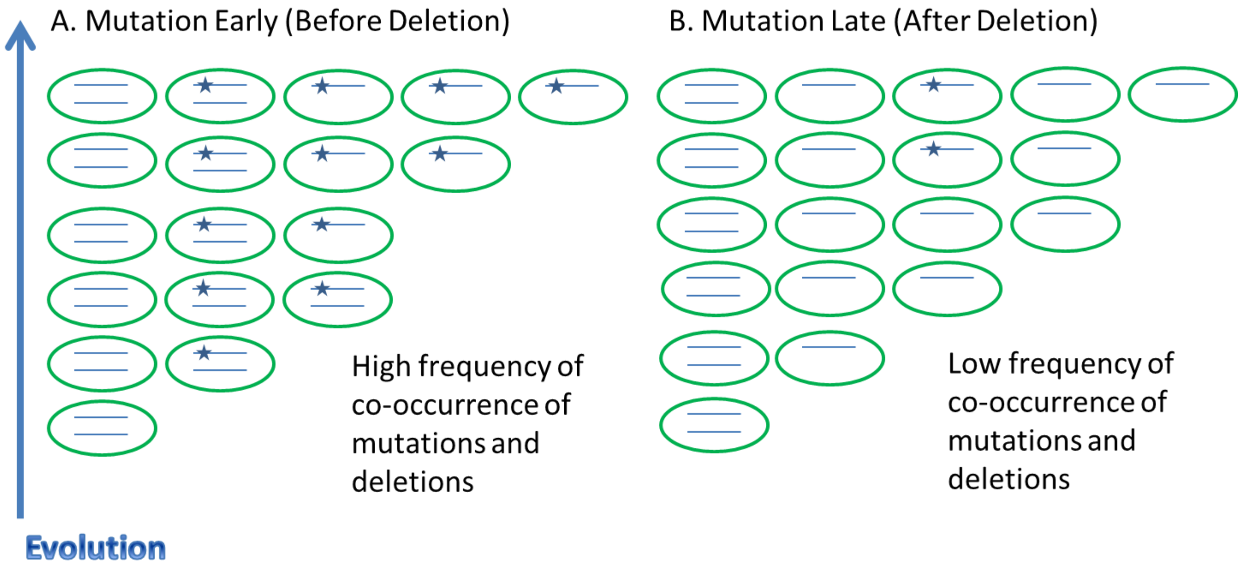

Mean mutant allele frequency ~ purity-corrected copy number frequency

Mean mutant allele frequency < purity-corrected copy number frequency

(A) Early mutation in a cancer gene, followed by the loss of a normal copy, will lead to higher mutant allele frequency relative to fraction of copy number loss. (B) Late mutation in cancer gene, subsequent to copy number loss, lead to lower fraction of chromosomes carrying the deleted mutation.

Figure S6. Genome-wide LOH and prevalence.

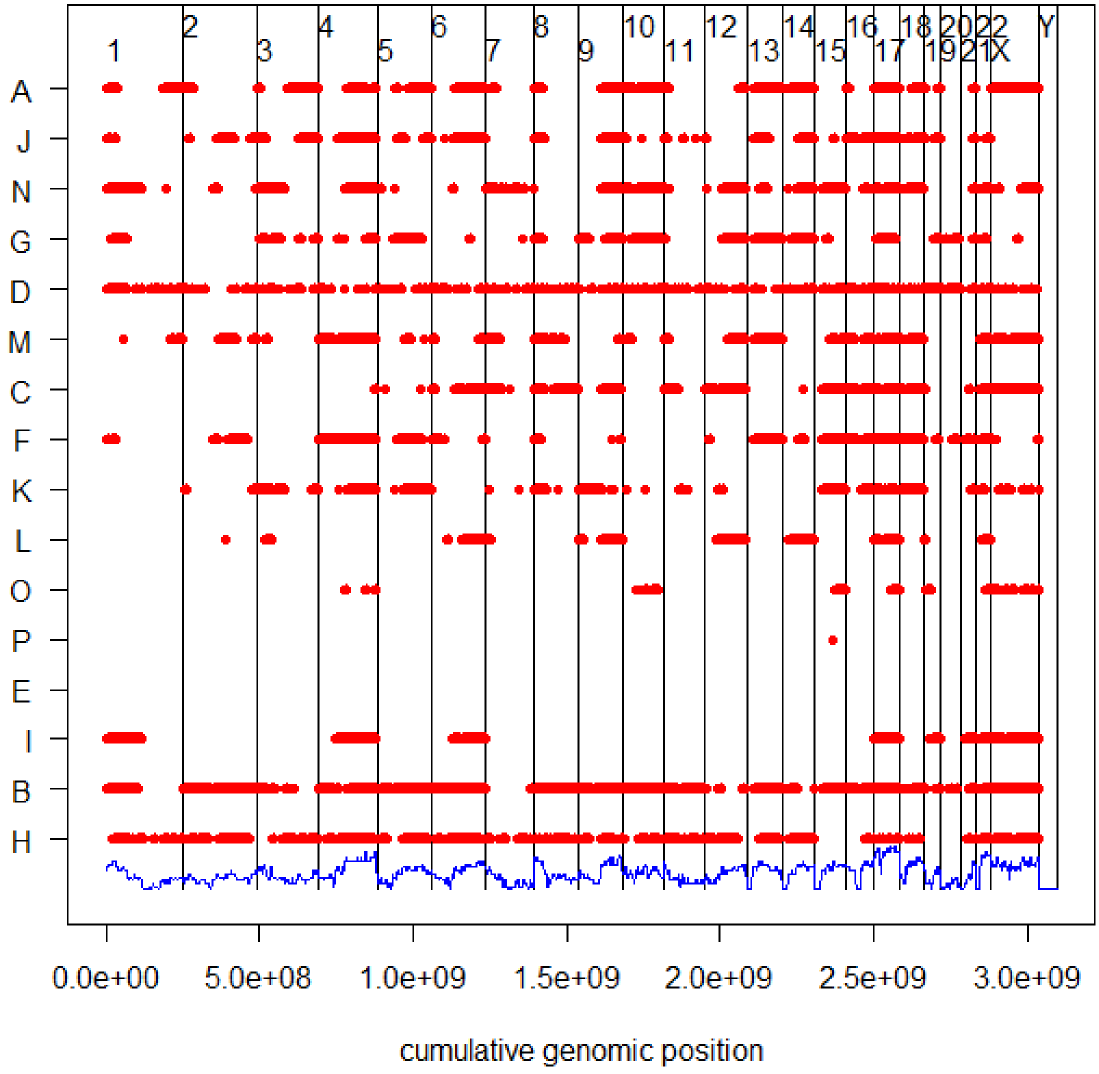

The bottom blue line shows the relative number of samples with LOH. Samples are ordered according to the clustering as shown in Figure S7.

Figure S7. Clustered genome-wide LOH.

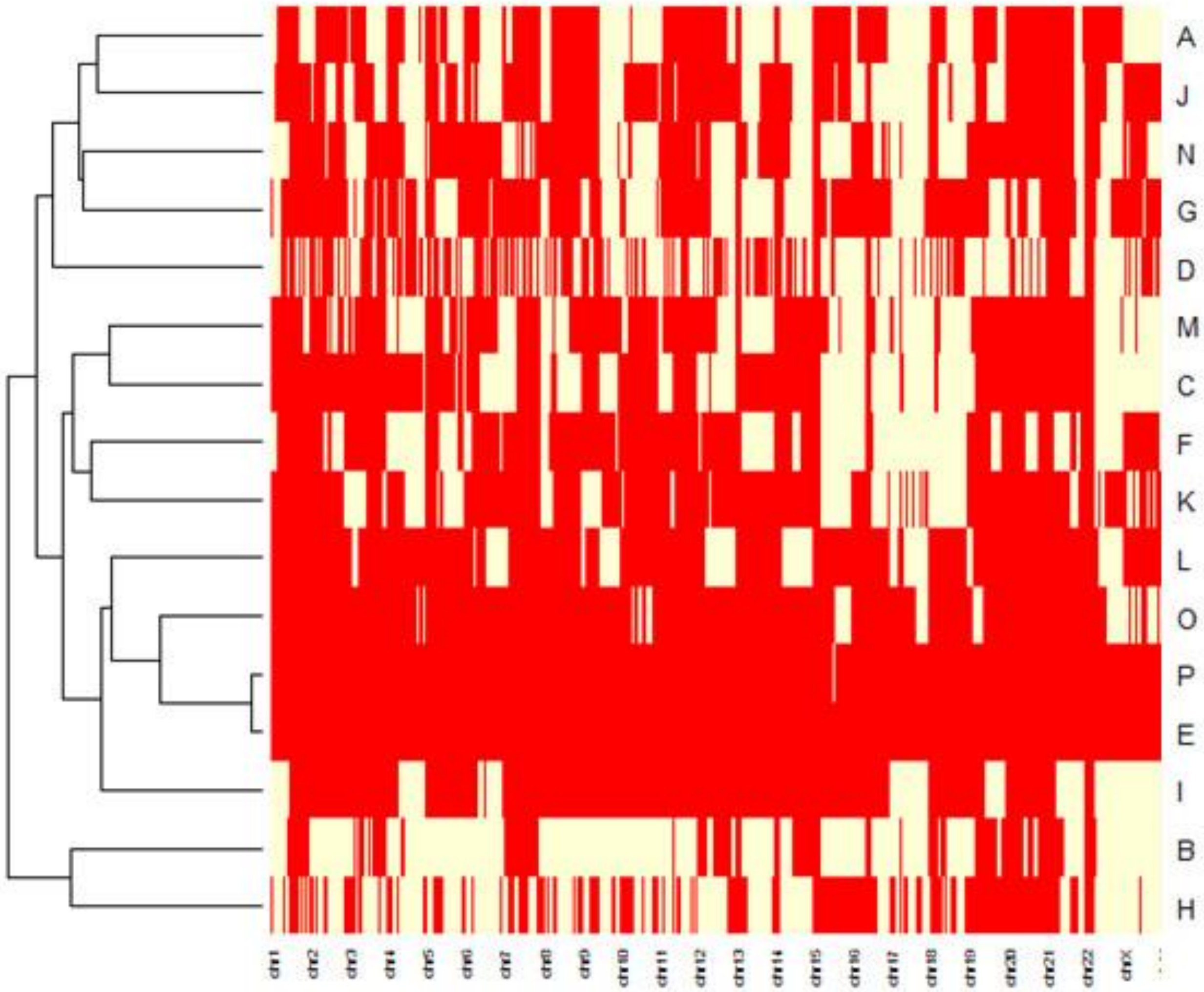

Heatmap is made by dividing each chromosome in 100 segments and assigning 0 if no LOH and 2 if an LOH. Dendrogram is computed using hiearchical clustering with default setting of the heatmap command in R. Yellow denotes regions with LOH.

Figure S8. **LOH segments per sample for selected chromosomes.**

A

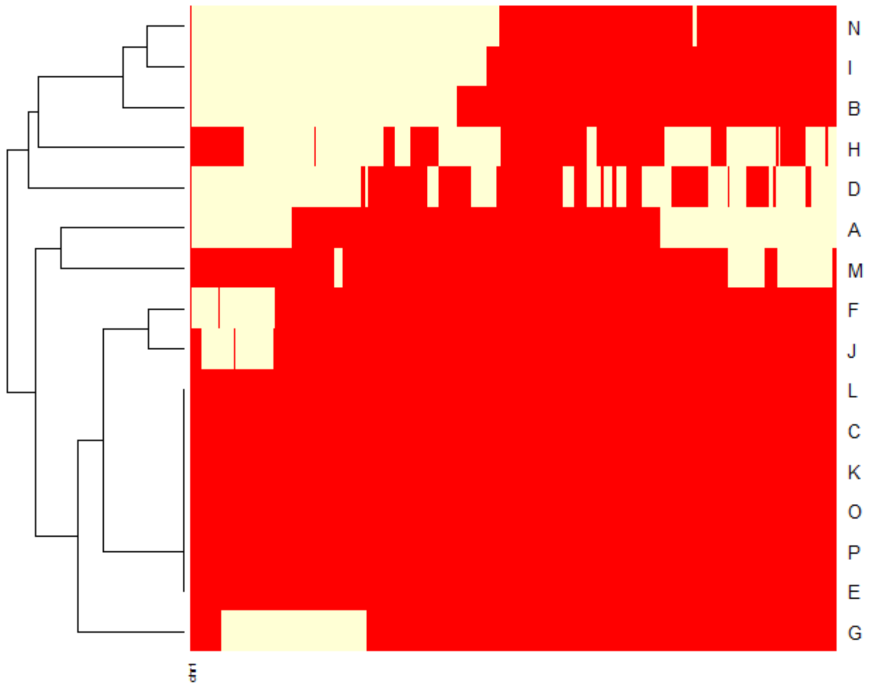

B

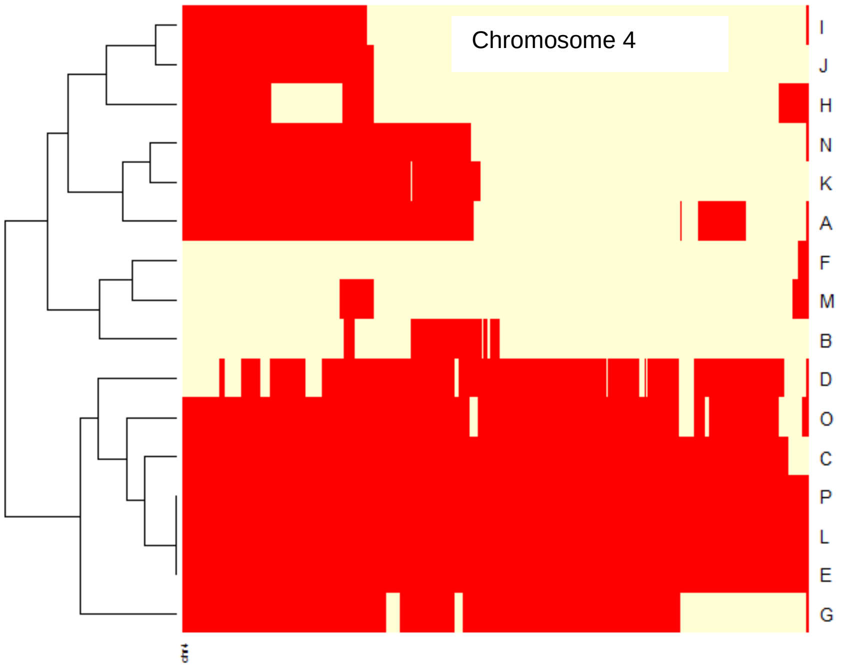

C

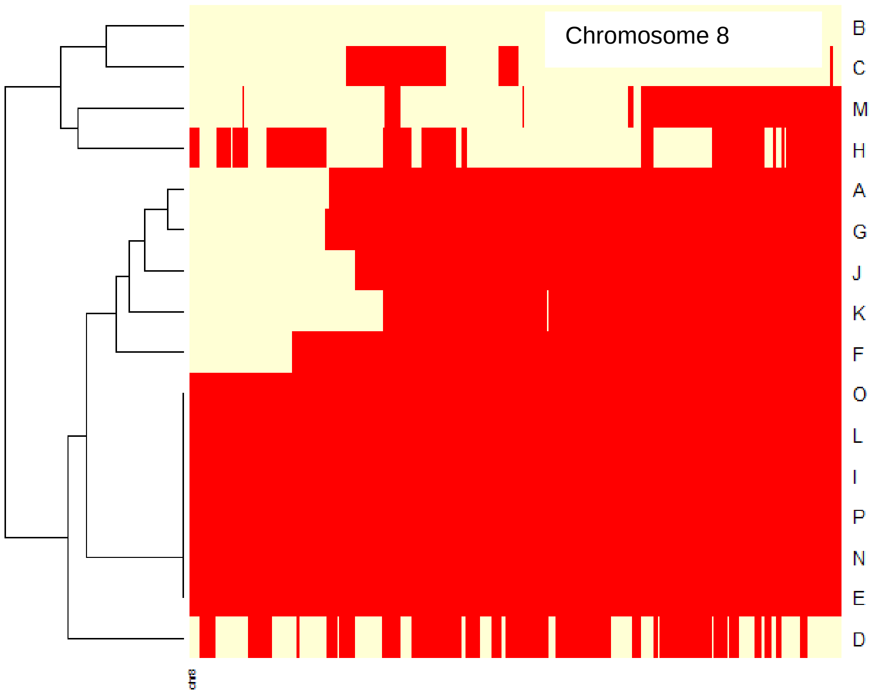
Chromosome 8
B
C
M
H
A
G
J
K
F
O
L
I
P
N
E
D
chr8

D

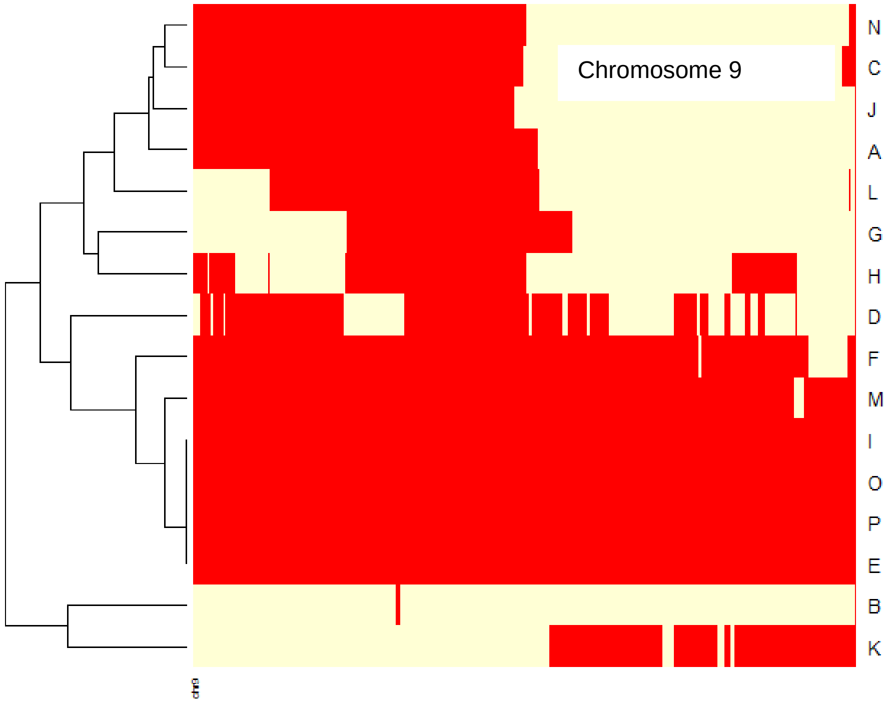
Chromosome 9
N
C
J
A
L
G
H
D
F
M
I
O
P
E
B
K
chr9

E

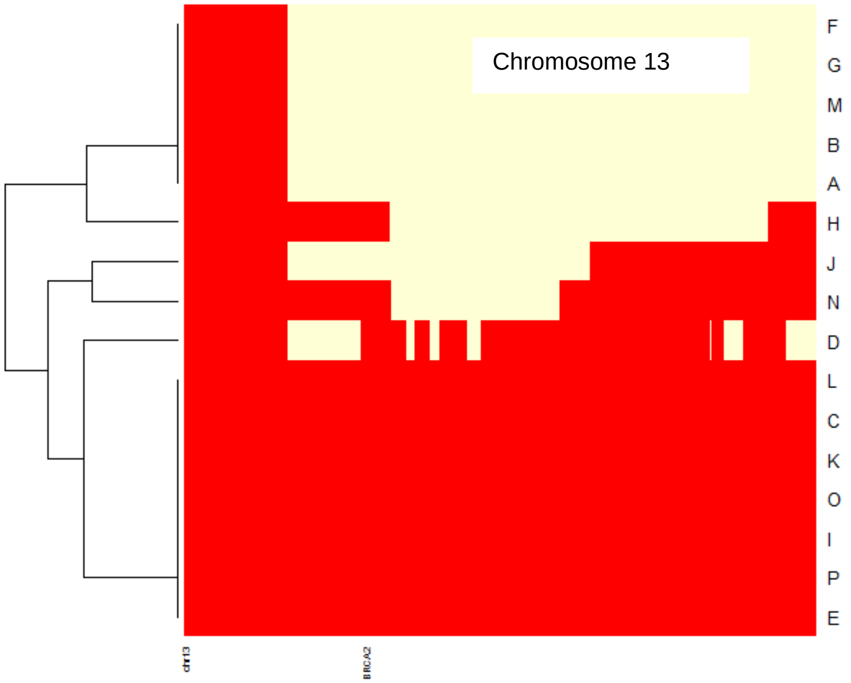
Chromosome 13
F
G
M
B
A
H
J
N
D
L
C
K
O
I
P
E
chr13
BRCA2

F

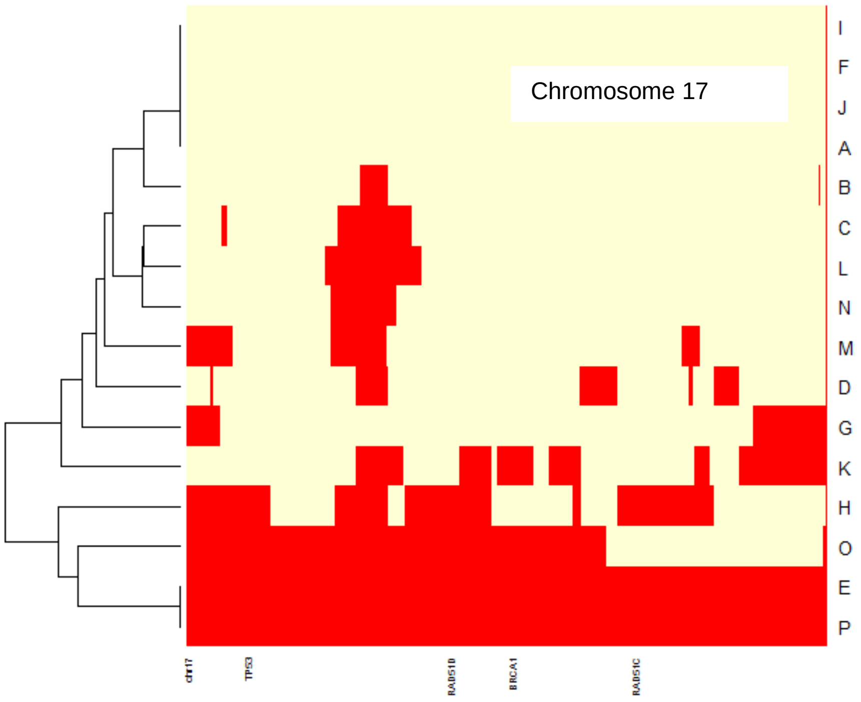
Chromosome 17
I
F
J
A
B
C
L
N
M
D
G
K
H
O
E
P
chr17
TP53
RAD51D
BRCA1
RAD51C

G

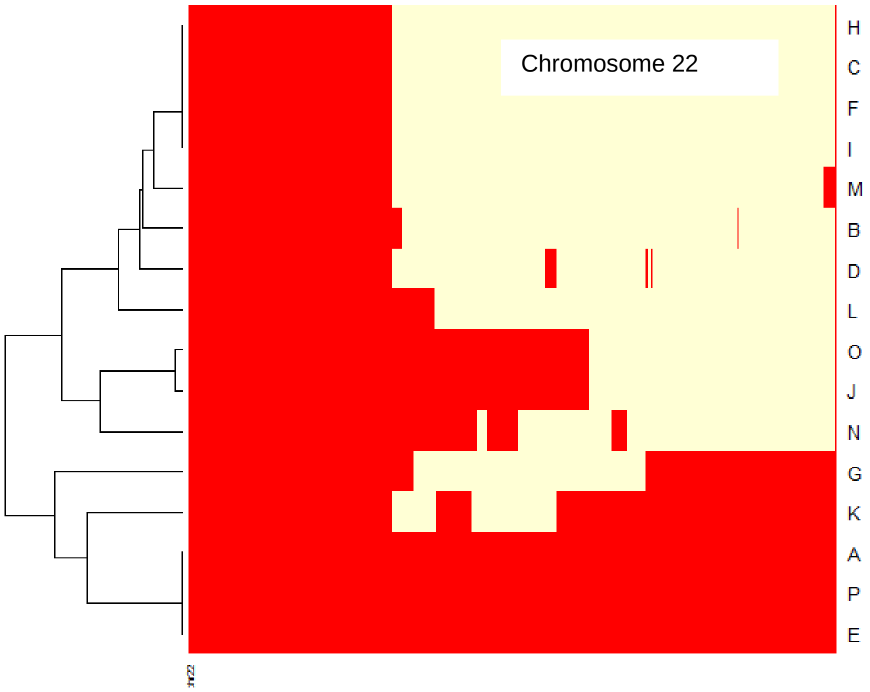

Dendrogram order is specific to each chromosome. (A) chromosome 1, (B) chromosome 4, (C) chromosome 8, (D) chromosome 9, (E) chromosome 13, (F) chromosome 17, (G) and chromosome 22.

Table S1. Characteristics of sequenced Mayo Clinic early-stage high-grade serous ovarian cancer cases (N=16).

ID	Diagnosis	Stage	Grade	Age at Diagnosis	Vital status	Follow up (months)	Tumor site	CA125	Tumor Nuclei	Megabase Sequenced (% Coverage) [Mean Coverage]			
										Tumor Exome	Normal Exome	Tumor Genome	Normal Genome
A	Ovarian Cancer	1A	3	52	Alive	208.651	right ovary	155	70%	62.6 (95.0)	62.2 (94.5)	2,624 (86.5) [43]	2,630 (86.7) [44]
B	Ovarian Cancer	1A	3	66	Alive	102.467	right ovary	42	90%	62.0 (95.3)	62.8 (95.4)	2,635 (86.9) [49]	2,640 (87.0) [46]
C	Ovarian Cancer	1B	3	82	Dead	73.586	bilateral ovaries	84	90%	61.7 (93.8)	62.1 (94.4)	2,622 (86.4) [42]	2,639 (87.0) [43]
D	Ovarian Cancer	1C	3	48	Alive	101.217	left ovary	1783	80%	58.8 (89.4)	62.2 (94.4)	2,584 (85.2) [46]	2,629 (86.7) [46]
E	Ovarian Cancer	1C	3	52	Alive	202.007	bilateral ovaries	87.4	70%	62.5 (95.0)	62.9 (95.6)	2,624 (86.5) [42]	2,632 (86.7) [43]
F	Ovarian Cancer	1C	3	60	Alive	85.987	left ovary	5	90%	61.6 (93.7)	59.6 (90.6)	2,631 (86.7) [50]	2,598 (85.6) [45]
G	Ovarian Cancer	1C	3	71	Alive	128.75	bilateral ovaries	175	70%	61.0 (92.7)	55.6 (84.5)	2,667 (87.9) [43]	2,575 (84.8) [50]
H	Ovarian Cancer	1C	3	50	Dead	60.461	left ovary	208	90%	62.3 (94.6)	62.2 (94.5)	2,630 (86.7) [43]	2,643 (87.1) [50]
I	Ovarian Cancer	2B	3	78	Dead	76.053	bilateral ovaries	9.7	90%	59.6 (90.5)	60.0 (91.1)	2,607 (85.9) [48]	2,623 (86.4) [54]
J	Ovarian Cancer	2B	3	50	Alive	80.132	left ovary	106.3	90%	59.7 (90.6)	62.6 (95.1)	2,594 (85.5) [48]	2,651 (87.4) [57]
K	Primary Peritoneal Cancer	2B	3	81	Dead	70.921	left ovary	12.2	70%	60.9 (92.5)	53.4 (81.2)	2,608 (85.9) [42]	2,521 (83.1) [45]
L	Ovarian Cancer	2C	3	50	Dead	97.467	bilateral ovaries	235.3	75%	60.4 (91.7)	63.2 (96.1)	2,642 (87.1) [49]	2,659 (87.7) [54]
M	Ovarian Cancer	2C	3	54	Alive	88.882	right ovary	763	90%	61.9 (94.1)	62.9 (95.5)	2,549 (84.0) [43]	2,604 (85.8) [47]
N	Ovarian Cancer	2C	3	55	Dead	112.105	bilateral ovaries	19	95%	63.2 (96.0)	62.7 (95.2)	2,616 (86.2) [45]	2,626 (86.6) [44]
O	Ovarian Cancer	2C	3	77	Dead	78.224	right ovary	29.8	70%	58.1 (88.2)	60.4 (91.8)	2,569 (84.7) [44]	2,621 (86.4) [52]
P	Ovarian Cancer	2C	3	84	Dead	53.059	right ovary	78	90%	63.4 (96.3)	63.3 (96.2)	2,627 (86.6) [43]	2,633 (86.8) [45]
									Mean:	61.3 (93.1)	61.1 (92.9)	2,614 (86.2) [45]	2,620 (86.4) [48]

Megabase sequenced and % coverage refer to regions with at least 10X coverage; mean coverage represents average coverage calculated by dividing the total bases sequenced with the size of haploid human genome.

Table S2. Genes in NextGen sequencing technical validation (N=42).

Region	Gene
chr1:45789914-45811142	*MUTYH*
chr2:215588275-215679428	*BARD1*
chr2:47595263-47715360	*MSH2 (+EPCAM)*
chr2:48005221-48039092	*MSH6*
chr3:10178319-10198744	*VHL*
chr3:142163078-142303668	*ATR*
chr3:37029979-37097337	*MLH1*
chr3:52430027-52449009	*BAP1*
chr4:84381094-84407290	*FAM175A (ABRA1)*
chr5:112038202-112186936	*APC*
chr5:131887630-131984595	*RAD50*
chr5:176331006-176434780	*UIMC1 (RAP80)*
chr7:142452319-142465927	*PRSS1*
chr7:152338589-152378250	*XRCC2*
chr7:6007870-6053737	*PMS2*
chr8:90940565-91001899	*NBN*
chr9:21962751-21999490	*CDKN2A*
chr10:43567517-43630795	*RET*
chr10:88511396-88689944	*BMPR1A*
chr10:89618195-89733532	*PTEN (+KILLIN)*
chr11:108088559-108244826	*ATM*
chr11:125494031-125528042	*CHEK1*
chr11:94145467-94232040	*MRE11A*
chr12:58137005-58151164	*CDK4*
chr13:32884617-32978809	*BRCA2*
chr14:104162954-104182823	*XRCC3*
chr14:68286496-69062738	*RAD51B*
chr15:40986378-41025356	*RAD51*
chr15:43698412-43803707	*TP53BP1*
chr16:23609483-23657678	*PALB2*
chr16:68766195-68874444	*CDH1*
chr17:33421811-33451888	*RAD51D*
chr17:41191313-41282500	*BRCA1*
chr17:56764963-56816692	*RAD51C*
chr17:59754985-59945755	*BRIP1*
chr17:7566720-7595863	*TP53*
chr18:20512839-20607449	*RBBP8 (CTIP)*
chr18:48550583-48616409	*SMAD4*
chr19:1200798-1233434	*STK11*
chr19:17377232-17391162	*BABAM1 (BRCC45) (MERIT40)*
chr22:29078731-29142822	*CHEK2*
chrX:154298695-154352349	*BRCC36*

Table S3. Results of NextGen sequencing technical validation (N=6).

Patient	Chrom	Start	Stop	Gene	Alleles	Functional Effect	Status in WGS sequencing
C	17	7578406	7578406	*TP53*	C>T	NM_000546:R175H<br>NM_001126115:R43H	somatic, valid in RNASeq
F	13	32936688	32936688	*BRCA2*	C>T	NM_000059:P2612S	somatic (26/40), valid in RNASeq(4/6)
F	17	7578263	7578263	*TP53*	G>A	NM_000546:R196stop<br>NM_001126115:R64stop	somatic, valid in RNASeq
L	17	7574003	7574004	*TP53*	-:G	frameshift	somatic, valid in RNASeq
M	17	7577062	7577063	*TP53*	-:T	frameshift	somatic, valid in RNASeq
P	13	32907487	32907489	*BRCA2*	-:TT	frameshift	germline heterozygous, somatic homozygous

Six samples were run on a candidate gene NextGen panel for technical validation of the mutations. All mutations found in this panel were also found in the whole genome sequencing.

Table S4. Genes in Sanger sequencing technical validation (N=47).

Chr	Gene	Number of Samples Sequenced
1	*CACNA1S*	3
1	*CHRM3*	3
1	*LRRC53*	3
2	*COL3A1*	3
2	*DNAH7*	4
2	*NEB*	3
2	*PIKFYVE*	3
2	*SLC5A7*	3
2	*TPO*	3
2	*TTN*	4
2	*XIRP2*	3
3	*MFN1*	3
3	*MUC4*	7
3	*PIK3CA*	3
3	*ZNF385D*	3
4	*KIAA1109*	3
5	*NIPBL*	3
5	*SDHA*	4
6	*GPR115*	3
6	*PKHD1*	3
7	*CACNA2D1*	3
7	*GRM3*	3
7	*MLL3*	3
8	*CNBD1*	3
8	*USP17L1P*	3
10	*ANK3*	3
10	*ANKRD30A*	3
10	*KNDC1*	3
10	*WDFY4*	3
11	*FAT3*	3
11	*MUC2*	4
11	*OR4A47*	3
12	*ADAMTS20*	3
12	*LRRIQ1*	3
12	*SLC16A7*	3
13	*BRCA2*	3
13	*MYO16*	3
13	*SACS*	3
14	*MYH6*	3
16	*ATBF1*	3
16	*CDH11*	3
19	*HNRNPUL1*	3
19	*MUC16*	3
21	*TIAM1*	4
X	*IL13RA1*	3
X	*NHS*	3
X	*THOC2*	3

Table S5. Estimated tumor purity and ploidy.

Sample	Percent Tumor Nuclei	N Somatic Variants used in Calculation of Purity	Purity (from Somatic Mutations)	GenoCN tumor purity estimates	WaveCNV Purity (from CNV and germline variants in blood and tumor)	WaveCNV estimated Ploidy
**A**	70%	193	0.63	0.506	0.76	1.7
**B**	90%	407	0.51	0.795	0.18	1.8
**C**	90%	563	0.79	0.507	0.77	3.4
**D**	80%	15	0.76	0.795	0.10	1.8
**E**	70%	245	0.65	0.506	0.74	**5.2**
**F**	90%	367	0.76	0.507	0.85	1.7
**G**	70%	376	0.59	0.507	0.78	1.9
**H**	90%	75	0.80	0.795	0.08	1.5
**I**	90%	119	0.83	0.507	0.83	1.8
**J**	90%	237	0.85	0.507	0.91	1.8
**K**	70%	157	0.80	0.795	0.39	2.1
**L**	75%	530	0.86	0.898	0.87	2.9
**M**	90%	691	0.87	0.506	0.85	2.6
**N**	95%	406	0.81	0.506	0.78	2.6
**O**	70%	276	0.66	0.507	0.74	**3.6**
**P**	90%	629	0.76	0.506	0.78	**5.1**

Computationally estimated tumor purity (percentage tumor cells in sample) estimated based on mutant allele frequency. Generally, the estimates are a lower than the pathology estimates, perhaps due to the presence of subclones or false positive variants; bold indicates high estimated ploidy (>3.5).

Table S6. Genotype concordance between Mayo Clinic sequence data and SNP array genotypes.

ID	Genotyping Concordance	
	Tumor	Normal
C	97.69%	99.46%
D	98.40%	99.34%
F	98.93%	99.03%
G	97.64%	98.19%
H	99.33%	99.32%
I	99.13%	NA
K	97.88%	97.85%
L	97.76%	NA
O	97.27%	99.29%

Total bases (in billion) mapped for the whole genome; NA, not available.

Table S7. Mutation count by sample and mutation types.

Patient	C->A	C->G	C->T	T->A	T->C	T->G	Mean	Total
A	1,344	845	**1,875**	1,133	1,146	**544**	1,148	6,887
B	762	517	**1,078**	818	1,014	**369**	760	4,558
C	923	521	**1,966**	969	1,480	**539**	1,066	6,398
D	228,738	6,500	**419,940**	**27,871**	191,080	157,615	171,957	1,031,744
E	2,133	1,299	**2,550**	1,980	2,229	**852**	1,841	11,043
F	1,194	659	**1,777**	1,287	1,340	**503**	1,127	6,760
G	2,012	1,678	**2,655**	1,538	1,945	**923**	1,792	10,751
H	194	135	**591**	312	573	**151**	326	1,956
I	831	413	**1,715**	1,195	1,122	**387**	944	5,663
J	1,221	1,102	**1,618**	1,143	1,293	**567**	1,157	6,944
K	946	561	**2,283**	1,368	1,595	**532**	1,214	7,285
L	968	601	**1,633**	890	1,106	**469**	945	5,667
M	2,934	2,943	**3,813**	2,356	2,752	**1,183**	2,664	15,981
N	3,195	1,897	**3,685**	2,889	2,789	**1,267**	2,620	15,722
O	897	496	**1,776**	1,162	1,203	**474**	1,001	6,008
P	3,694	3,220	**4,485**	2,943	3,214	**1,425**	3,164	18,981
**Median %**	18%	11%	**27%**	16%	20%	**8%**		
Paired t-test vs Mean	0.14391	0.00053	7.08E-08	0.76091	1.43E-05	8.08E-06		

Number of types of mutations (reverse-Complement mutations merged); C->T transitions dominate and T->G are suppressed; bold indicates most and least frequent mutation type per sample.

Table S8. Significantly mutated genes in Mayo Clinic and TCGA early stage HGS cases.

Gene	Indels	SNVs	Covered Bps	Mutations per Mbp	P-value
TP53	9	20	54130	535.75	<10E-10
TPSAB1	0	3	6599	454.61	0.0001
VN1R1	0	2	37835	52.86	0.0010
AMIGO2	0	3	58835	50.99	0.0121
FOXD4L1	0	2	15655	127.75	0.0336
IFNA21	0	2	21035	95.08	0.0493
TPO	0	3	124657	24.07	0.0238
MEF2BNB-MEF2B	1	1	44503	44.94	0.0484
CHRM3	0	3	63035	47.59	0.0359
RFPL3	0	2	37864	52.82	0.0737
XIRP2	1	3	441539	9.06	0.0252
TDP1	2	0	88725	22.54	0.0643
GPR112	0	4	382730	10.45	0.0385
TPK1	0	2	42280	47.3	0.0967
SLPI	0	2	25340	78.93	0.1734
MAP2	0	3	225470	13.31	0.0703
GDF3	0	2	42070	47.54	0.1249
SPPL2A	0	2	76272	26.22	0.1574
CRB1	0	3	176820	16.97	0.1181
UNC5D	0	3	130795	22.94	0.1215
MC2R	0	2	33635	59.46	0.1751
JUP	1	1	108592	18.42	0.1707
FAM55D	0	2	66885	29.9	0.1942
P2RY13	0	2	37835	52.86	0.1951
VEGFC	0	2	53662	37.27	0.1843
ZNF804A	0	2	135870	14.72	0.2019
CCDC134	0	2	37945	52.71	0.2339
EFEMP1	0	2	82702	24.18	0.2062
CDSN	0	2	57896	34.54	0.2362
SEL1L2	0	2	118300	16.91	0.2281
AIM2	0	2	49385	40.5	0.2437
B4GALT5	0	2	59159	33.81	0.2430
SLC5A7	0	2	80068	24.98	0.2597
LRRC2	0	2	59080	33.85	0.2830
ASCC3	0	3	332186	9.03	0.1840
BCAR1	0	2	81666	24.49	0.2854
GOPC	0	2	69737	28.68	0.3103
CNOT3	0	2	111749	17.9	0.2824
TARBP1	0	2	198494	10.08	0.2872
CNTNAP2	0	2	200009	10	0.3163
MKL1	0	2	116724	17.13	0.3340
DNTT	0	2	80185	24.94	0.3682
NEIL3	0	2	88074	22.71	0.3678
NUP210L	0	2	289202	6.92	0.2695
COL4A4	0	3	275213	10.9	0.2570
DYNC1I1	0	2	101343	19.73	0.3822
GTPBP1	0	2	85907	23.28	0.3688
HRNR	1	1	189666	10.54	0.3145

Gene	Indels	SNVs	Covered Bps	Mutations per Mbp	P-value
LLGL2	0	2	146010	13.7	0.3515
MEST	0	2	74343	26.9	0.3463
MME	0	2	126732	15.78	0.3613
RCC1	0	2	81596	24.51	0.3612
TNFRSF8	0	2	98103	20.39	0.2917
THOC2	0	3	254681	11.78	0.2316
CUX1	0	3	222131	13.51	0.2760
EPB41L3	0	2	155614	12.85	0.3630
NEK4	0	2	109746	18.22	0.4324
PTPRZ1	0	3	317443	9.45	0.3155
WDR49	0	2	101290	19.75	0.4219
ADAMTS4	0	2	105039	19.04	0.4223
ANUBL1	0	2	101114	19.78	0.4215
PEG3	0	2	175272	11.41	0.3961
HCN4	0	2	85517	23.39	0.4515
DSG3	0	2	151295	13.22	0.4078
KCNT2	0	2	163345	12.24	0.4301
BRCA2	0	3	422955	7.09	0.3129
CSRP2BP	0	2	108710	18.4	0.4405
TGM5	0	2	118001	16.95	0.4664
GPR179	0	3	258408	11.61	0.3661
RIMS2	0	2	171389	11.67	0.4300
DCC	0	2	219035	9.13	0.4275
NLRP4	0	2	130515	15.32	0.4751
ADAMTS3	0	2	180906	11.06	0.5400
CD163L1	0	2	196175	10.19	0.4276
GOLGB1	0	3	387100	7.75	0.3756
CACNA2D1	0	2	190338	10.51	0.4818
OTUD4	0	2	155633	12.85	0.5437
TGFBR3	0	2	126560	15.8	0.5249
FAT2	0	3	502898	5.97	0.5699
CHD6	0	2	368340	5.43	0.6075
AGL	0	2	234605	8.52	0.6448
RNF213	0	2	586215	3.41	0.7360
SHROOM2	0	2	171344	11.67	0.6692
KDR	0	2	196712	10.17	0.5000
ATP10B	0	2	201880	9.91	0.5098
MLL3	0	3	636213	4.72	0.5073
SPTA1	0	2	377265	5.3	0.8366
MYH1	0	2	321405	6.22	0.5963
MTOR	0	2	436868	4.58	0.8877
NIPBL	0	2	383366	5.22	0.6214
SEC31A	0	2	193677	10.33	0.6014
ADAMTS17	0	2	152172	13.14	0.6073
ODZ1	0	2	364721	5.48	0.6408
USH2A	0	2	697007	2.87	0.6950
ADAMTS12	0	2	239855	8.34	0.6300
DYNC1H1	0	2	661523	3.02	0.9422
SH3TC2	0	2	176608	11.32	0.6498

Gene	Indels	SNVs	Covered Bps	Mutations per Mbp	P-value
MACF1	0	2	1005585	1.99	0.7686
MYO16	0	2	234130	8.54	0.6764
COL7A1	1	2	605245	4.96	0.4262
BAZ2B	0	2	300568	6.65	0.7075
KIF1B	0	2	338003	5.92	0.7195
SBF2	0	2	269323	7.43	0.7228
PIK3C2B	0	2	240437	8.32	0.7446
COL4A5	0	2	293427	6.82	0.7530
HIVEP3	0	2	251409	7.96	0.7653
COL11A1	0	2	341597	5.85	0.7908
CACNA1S	0	2	282006	7.09	0.7952
ZFHX3	0	2	363162	5.51	0.8170
PKD1L1	0	2	426264	4.69	0.8443
DNAH7	0	2	565962	3.53	0.8717
HMCN1	0	2	859600	2.33	0.9605
SYNE2	0	2	992947	2.01	0.9543
VPS13C	0	2	569181	3.51	0.8654

Only genes with at least three mutations are shown; Indels, Number of Insertion/Deletion variants in the region/gene; SNVs, Number of Single Nucleotide Variants; Covered Bps, gene size; Mutations per Mbp, Mutations per Megabases; P-value, p-value from Fisher's combined p-value test; mutations were also found in the following genes known to be polymorphic: *HLA-DQA1, OR8B3, OR2A14, OR8H3, OR4A47,* and *OR4E2.*

Table S9. Structural Variant types and counts

Patient	Inter-chromosomal Translocations (CTX)	Deletions	Insertions	Intra-chromosomal Translocations (ITX)	Tandem Duplications (INV)	Total SV	*WaveCNV estimated Ploidy*	*HRD Score*	*LOH Burden*
**A**	1	1	20	0	0	22	***1.7***	*27*	*0.43*
**B**	0	1	1	8	0	10	*1.8*	*36*	*0.68*
**C**	5	4	9	21	0	39	*3.4*	*21*	*0.33*
**D**	0	0	0	0	0	0	*1.8*	*4*	*0.39*
**E**	0	2	0	1	0	3	**5.2**	*0*	*0*
**F**	3	2	12	6	0	23	*1.7*	*20*	*0.33*
**G**	1	2	5	0	0	8	*1.9*	*22*	*0.31*
**H**	0	0	0	0	0	0	*1.5*	*26*	*0.54*
**I**	2	2	5	2	0	11	*1.8*	*15*	*0.21*
**J**	21	12	41	1	0	75	*1.8*	*20*	*0.34*
**K**	2	6	12	12	0	32	*2.1*	*14*	*0.25*
**L**	3	3	9	2	0	17	*2.9*	*10*	*0.16*
**M**	14	19	21	0	0	54	*2.6*	*25*	*0.36*
**N**	6	16	15	3	0	40	*2.6*	*25*	*0.36*
**O**	6	5	0	10	0	21	***3.6***	*8*	*0.08*
**P**	3	3	1	0	0	7	**5.1**	0	0.0006

Structural Variation events as predicted by CREST. No apparent association with HRD score, ploidy, or LOH burden. SV with start/stop within 15bp (duplicates) were removed from the count. Crest SV type “INS” are actually Tandem duplications.

Table S10. Recurrent genes in SV

# of samples	chrom	gene_start	gene_stop	gene
3	chr3	173116243	173999091	NLGN1
2	chr1	215796235	216596556	USH2A
2	chr1	216676587	217311009	ESRRG
2	chr1	217603833	217804311	GPATCH2
2	chr1	217804694	218040364	SPATA17
2	chr1	218458628	218504431	RRP15
2	chr1	218517537	218518945	LOC728463
2	chr1	218518675	218614702	TGFB2
2	chr1	219254316	219346812	LOC643723
2	chr1	219347191	219385068	LYPLAL1
2	chr1	220046618	220292760	RNU5F-1
2	chr1	220087605	220101780	SLC30A10
2	chr1	220141941	220219728	EPRS
2	chr1	220230823	220263028	BPNT1
2	chr1	220267454	220320975	IARS2
2	chr1	220291194	220291195	MIR215
2	chr1	220291498	220291499	MIR194-1
2	chr1	220321609	220445677	RAB3GAP2
2	chr1	220373883	220373884	MIR664
2	chr1	220373887	220373888	SNORA36B
2	chr1	220439520	220439521	AURKAPS1
2	chr1	220701567	220835506	MARK1
2	chr1	220863627	220870071	C1orf115
2	chr1	220921675	220957261	MARC2
2	chr1	220960038	220986758	MARC1
2	chr1	221052742	221058044	HLX
2	chr12	99128568	100378013	ANKS1B
2	chr19	10764936	10799986	ILF3
2	chr19	9759337	9785691	ZNF562
2	chr3	123813557	124438315	KALRN
2	chr3	168801286	169381158	MECOM
2	chr3	169557028	169587593	LRRC31
2	chr3	169629481	169656292	SAMD7
2	chr3	170714136	170744457	SLC2A2
2	chr3	170780291	171177850	TNIK
2	chr3	170824452	170824453	MIR569
2	chr3	171757417	172115263	FNDC3B
2	chr4	147628178	147866861	TTC29
2	chr4	40198526	40245580	RHOH
2	chr4	40337345	40356535	CHRNA9
2	chr4	44175919	44450538	KCTD8
2	chr4	56298659	56412949	CLOCK
2	chr4	73146685	73434477	ADAMTS3
2	chr4	73940501	74124383	ANKRD17
2	chr4	74269971	74286809	ALB
2	chr4	77234191	77328375	CCDC158
2	chr4	86396283	86921873	ARHGAP24

2	chr6	107811316	107979411	SOBP
2	chr6	24705089	24718894	C6orf62
2	chr6	36708554	36807151	CPNE5
2	chr6	41765382	41863062	USP49
2	chr6	44796469	45345505	SUPT3H
2	chr6	53362139	53409441	GCLC
2	chr6	62389864	62995851	KHDRBS2
2	chr6	73331570	73905135	KCNQ5
2	chr6	74405507	74533355	CD109
2	chr8	37716464	37756957	RAB11FIP1
2	chr8	38854504	38961217	ADAM9
2	chr8	38965049	39142316	ADAM32

Genes in SV events present in at least one sample.

Structural Variation events as predicted by CREST. Many SV tend to appear in Clusters.309/543 (57%) of SV are clustered. SV similar (with start/stop within 15bp) to another SV are annotated as duplicate.

Table S11. Functional annotation enriched by genes with recurrent SV

	Annotation cluster	Enrichment score	Count	P_Value
GOTERM_CC_FAT	cell projection	1.58	7	0.0059
GOTERM_BP_FAT	embryonic morphogenesis	1.3	4	0.041
GOTERM_MF_FAT	GTPase regulator activity	1.29	5	0.025
